# Cortical structural differences in major depressive disorder correlate with cell type-specific transcriptional signatures

**DOI:** 10.1038/s41467-021-21943-5

**Published:** 2021-03-12

**Authors:** Jiao Li, Jakob Seidlitz, John Suckling, Feiyang Fan, Gong-Jun Ji, Yao Meng, Siqi Yang, Kai Wang, Jiang Qiu, Huafu Chen, Wei Liao

**Affiliations:** 1 The Clinical Hospital of Chengdu Brain Science Institute, School of Life Science and Technology, University of Electronic Science and Technology of China, Chengdu, P.R. China; 2 MOE Key Lab for Neuroinformation, High-Field Magnetic Resonance Brain Imaging Key Laboratory of Sichuan Province, University of Electronic Science and Technology of China, Chengdu, P.R. China; 3 Children’s Hospital of Philadelphia, Department of Child and Adolescent Psychiatry and Behavioral Science, Philadelphia, PA USA; 4 University of Pennsylvania, Department of Psychiatry, Philadelphia, PA USA; 5 University of Cambridge, Department of Psychiatry, Cambridge, UK; 6 Department of Medical Psychology, Chaohu Clinical Medical College, Anhui Medical University, Hefei, P.R. China; 7 School of Psychology, Southwest University, Chongqing, P.R. China

**Keywords:** Gene expression profiling, Depression, Brain

## Abstract

Major depressive disorder (MDD) has been shown to be associated with structural abnormalities in a variety of spatially diverse brain regions. However, the correlation between brain structural changes in MDD and gene expression is unclear. Here, we examine the link between brain-wide gene expression and morphometric changes in individuals with MDD, using neuroimaging data from two independent cohorts and a publicly available transcriptomic dataset. Morphometric similarity network (MSN) analysis shows replicable cortical structural differences in individuals with MDD compared to control subjects. Using human brain gene expression data, we observe that the expression of MDD-associated genes spatially correlates with MSN differences. Analysis of cell type-specific signature genes suggests that microglia and neuronal specific transcriptional changes account for most of the observed correlation with MDD-specific MSN differences. Collectively, our findings link molecular and structural changes relevant for MDD.

## Abstract (web summary)

The correlation between brain structural changes in major depressive disorder (MDD) and gene expression is unclear. Here, the authors explore the correlation between cell type-specific gene expression changes and cortical structural difference in individuals with major depressive disorder.

## Introduction

Major depressive disorder (MDD) is a prevalent worldwide psychiatric disease that often first occurs in adolescence^1^. Despite significant efforts, our current understanding of its pathophysiology is unclear with inconsistent brain architectural changes^2^ and the variable effects of treatment^3^. Although neuroimaging studies show some focal structural alterations^4^, functionally MDD is increasingly recognized as a disorder involving brain “disconnectivity”^5^.

Recent magnetic resonance imaging (MRI) studies indicate that abnormal structural connectomes primarily involve frontal-limbic regions, including the dorsolateral prefrontal cortex, the anterior cingulate cortex, posterior cingulate cortex/precuneus, and orbitofrontal cortex^6,7^. Investigating MDD brain structural connectomes has primarily relied on two approaches: identifying the white-matter networks by diffusion-weighted imaging (DWI) tractography, and structural covariance networks of between-subject correlations of morphological measures^6,8–10^. Although some studies have reported reduced white-matter connectivity in diverse subnetworks^9^, DWI tractography remains challenging, especially in estimating the connectivity strength of long-distance projections^11^. Structural covariance analysis has shown significant changes associated with depression, particularly segregation^12^. However, this technique relies for its accuracy on large sample sizes, and generally cannot be used for individual analysis. Its biological interpretation also remains controversial^13^.

Morphometric similarity network (MSN) analysis has been a recent major step forward in revealing macroscale cortical organization^14^. Rather than measuring the interregional correlation of a single MRI feature across participants, MSNs capture the interregional correlation of multiple morphometric features from multiple modalities in a single individual. Methodologically, MSNs can be constructed for individuals and potentially have closer associations with cytoarchitectonic classes, distinguished by cortical lamination patterns, compared with DWI tractography. In addition, Seidlitz et al. reported three biological associations of MSNs^14^. First, strongly connected cortical areas have high levels of co-expressed genes. Second, strongly connected cortical areas often belong to the same cytoarchitectonic class, supported by histological evidence from nonhuman primates^15,16^. Third, cortical regions that are more morphometrically similar are likely to be axonally connected to each other^17^. Finally, clinical abnormalities of the MSN in individuals with schizophrenia are highly associated with brain expression of schizophrenia-related genes^18^, and uncover transcriptomic and cellular profiles of regional brain vulnerability to neurogenetic disorders^19^. Although MSNs are a reliable and robust method, their use for uncovering morphometric differences in MDD remains untried.

Genetic factors play important roles in brain connectomes^20,21^, and brain-wide gene expression atlases bridge the gap between connectomes and transcriptomes^22^. The Allen Human Brain Atlas (AHBA) microarray dataset has been used to identify transcriptomes associated with human neuroimaging^23,24^ with multimodal evidence suggesting a link between conserved gene expression and functionally relevant circuitry^25–28^. Combining neuroimaging and gene transcripts has provided insight into how disease-related alterations at the microscale architecture drive macroscale brain abnormalities in various mental disorders^18,19,29–31^. More recently, Anderson et al. linked cortical thickness and functional correlations in MDD to AHBA expression data, revealing dysregulation of somatostatin interneurons and astrocytes^32^.

In this work, we link MDD-related MSN abnormalities and transcriptional data to advance our understanding of the relationship of molecular mechanisms to structural changes in depression. First, we describe the moderately replicable MDD-related MSN abnormalities in two independent cohorts. Second, we establish the relationship between MDD-related changes in MSN and anatomically patterned gene expressions using the AHBA to obtain MDD-related genes. Based on the postmortem samples from individuals with MDD, differential gene expressions (DGEs), closely linked to transcriptionally upregulated genes, are spatially correlated with MDD-related MS differences. Third, we perform a functional enrichment analysis to infer the ontological pathways of MDD-related genes which converge with synapse-related terms. Fourth, we link abnormal MSN-related genes to cell types, specifying microglia and neurons as contributing most to the transcriptomic relationship of MDD-related changes in MSN. These observations help us understand how brain-wide gene expression and cell types capture molecularly validated anatomical differences in MDD.

## Results

### Experimental design

**Fig. 1:**
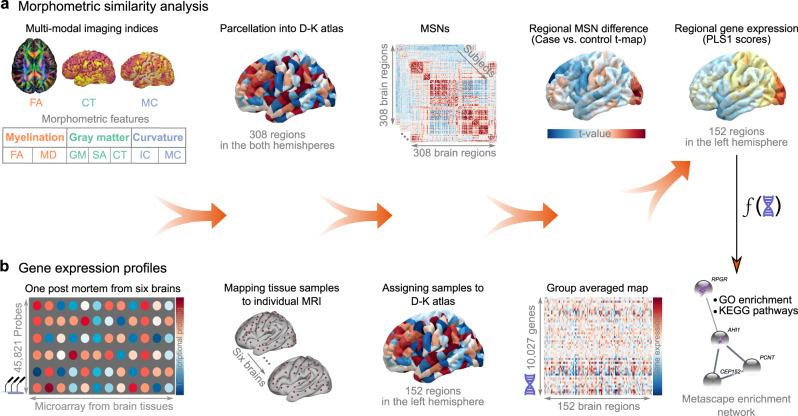
Study overview. **a** Morphometric similarity network (MSN) analysis. Individual MSNs were constructed across multimodal MRI features (e.g., myelination, gray matter, and curvature) to produce a 308 × 308 matrix (depicted by a subdivision of the Desikan–Killiany atlas, D–K 308). Then, MDD-HC (case-control) differences across regions were computed. **b** Gene expression profiles. Gene expression profiles from the Allen Human Brain Atlas in 152 regions (left hemisphere only) were averaged across six postmortem brains. Partial least squares (PLS) regression was then used to identify imaging-transcriptomic associations. Finally, enrichment analysis was performed on the gene list associated with the first component of PLS (PLS1).

This study combined multimodal neuroimaging and transcriptomics data to determine links between gene expression and changes in the MSN of individuals with MDD relative to healthy controls (HC) (Fig. 1). We included two independent cohorts: a discovery cohort and a replication cohort. Specifically, the replication cohort was used to validate the case-control changes in MSN and transcriptional enrichment pathways. Quality control of the images excluded 65 participants: 217 individuals with MDD and 208 HC in the discovery cohort, and 42 individuals with MDD and 35 HC in the replication cohort were used for subsequent analyses (Table S1). We report the results based on the discovery cohort, unless stated otherwise. There were no significant (*p* > 0.05) between-group differences in the means of image quality, age, and sex (Supplemental Result 1 and Fig. S1). Age and sex were used as covariates in linear models of between-group differences to reduce model error.

### MDD-related changes in MSN

**Fig. 2:**
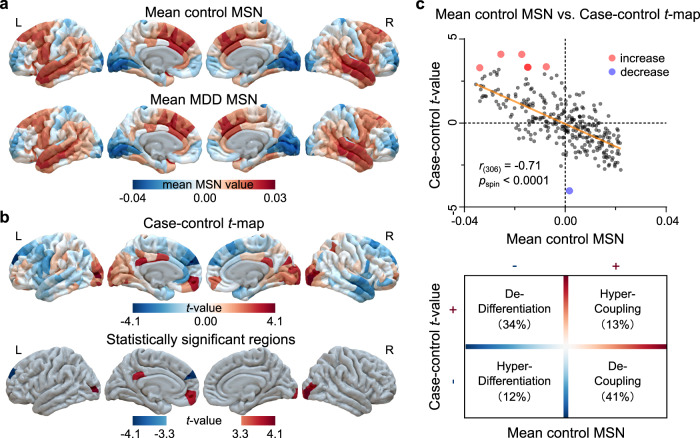
Case-control differences of regional morphometric similarities. **a** Mean regional morphometric similarity network (MSN) pattern of control subjects and individuals with MDD. The frontal and temporal lobes exhibited high MSN values, whereas the occipital and somatosensory cortices showed low MSN values. **b** Case-control comparison (*t*-map) of regional MSN (first row, unthresholded). Seven cortical regions showed statistically significant differences (bottom row, *p*_FDR_ < 0.05). **c** A scatterplot of the mean regional MSN (*x*-axis) and case-control *t*-map (*y*-axis) (first row) (Pearson’s *r*_(306)_ = −0.71, *p*_spin_ = 0.00). *p* value was not corrected for multiple comparisons, and was determined based on a one-sided test. Most cortical regions exhibited dedifferentiation (34%) and decoupling (41%) in individuals with MDD (bottom row).

We first calculated the MSN [Desikan–Killiany (D–K)-308 regions × 308 regions] from interregional Pearson’s correlation of seven features derived from T1 weighted (T1w) and DWI images acquired from each participant^14,18,19^. We then calculated the regional MSN values as the sum of correlation weights between a given region and all other regions in matrix (without thresholding)^18,19^. Within-group averaged summed weights created an anatomical distribution of positive and negative MSN correlations in HC (Fig. 2a) that were consistent with a previous report by Morgan et al.^18^ yielding a correlation of mean regional values, Pearson’s *r*_(306)_ = 0.91, *p*_spin_ < 0.0001 (Supplemental Result 2 and Fig. S2), which was significant after correction for multiple comparisons by spatial permutation testing (spin test)^33^. Threshold selection is a complex issue in network construction. To validate the thresholding effects^14^, we calculated the MSN at a range of connection densities (10‒90% in 10% increments). At all connection densities, the MSN maps demonstrated similar patterns with the mean HC MSN map at 100% connection density (i.e., no thresholding) (Supplemental Result 3 and Fig. S3).

Summing regional MSN weights across all regions for each participant, individuals with MDD did not differ from HC (Supplemental Result 4 and Fig. S4). Decomposed into regions, we obtained the case-control (MDD-HC) *t*-map by conducting a linear regression with age, sex, and education included as covariates and then extracting the two-sided mean *t*-statistic value comparing individuals with MDD and HC in each region. Positive and negative *t*-values denote increased and decreased MSN in individuals with MDD, respectively. MDD participants exhibited decreased MSN weights in the left superior frontal, and increased MSN weights in the left medial orbitofrontal (part2), isthmus cingulate cortex (part2), lateral occipital cortex (part7), and the right lateral occipital cortices (part 1, part 6, and part 8), when compared with HC (all *p*_FDR_ < 0.05; Fig. 2b; Supplemental Result 5 and Table S2). A decreased regional MSN in individuals with MDD implies decreased morphometric similarity (or greater morphometric differentiation) between these areas and the rest of the cortex, which is interpreted as reduced anatomical connectivity to and from the less similar, more differentiated cortical areas, and conversely for increased regional MSN^18^. The MDD-HC *t*-map was validated across a series of thresholded MSNs (10‒90% in 10% increments), demonstrating a minimal effect of threshold selection on MDD-related differences (Supplemental Result 6 and Fig. S5).

Because total intracranial volume (TIV) is an important factor for volumetric analyses of brain regions^34^, we also validated the effect of TIV on the MDD-HC *t*-map by including it as a covariate in the linear regression model (LRM) in the discovery cohort with minimal effect on MDD-related changes in MSN (Supplemental Result 7 and Fig. S6). In addition, we divided the individuals with MDD in the discovery cohort into two subgroups: drug-naive and drug-experienced patients, to explore the medication effects on MSNs. We found that irrespective of medication status, similar patterns to the case-control *t*-map (Fig. 2b) were observed (Supplemental Result 8 and Fig. S7).

Next, to make the findings generalizable to other levels of brain organization; namely, at a systems-level of brain functional networks composed of subnetworks that are observable during rest and are known to support cognitive function, and at a microstructural-level by characteristics that define the von Economo classes, brain regions were also assigned to each of the Yeo 7 functional networks (Supplemental Result 9 and Fig. S8a)^35^, and each of the von Economo cytoarchitectonic classes (Fig. S8b)^36^. Cross-sectionally, individuals with MDD exhibited increased MSN in the Yeo visual network (*p*_FDR_ = 0.007; Supplemental Table S3 and Fig. S8c). For the von Economo cytoarchitectonic classes, individuals with MDD had increased MSN in secondary sensory cytoarchitectonic class (*p*_FDR_ = 0.02; Supplemental Table S4 and Fig. S8d).

The case-control *t*-map was significantly spatially correlated with the mean control regional MSN: Pearson’s *r*_(306)_ = −0.71, *p*_spin_ < 0.0001 (Fig. 2c), indicating that more connected regions tend to show larger case-control differences^18,19^. Negative regional *t*-values and positive mean MSN represents decoupling in individuals with MDD relative to HC and was found in 34% of regions, whereas 41% of regions had positive *t*-values and negative mean MSN representing dedifferentiation in individuals with MDD relative to HC.

We assessed the relationship between case-control changes in MSN and symptoms using Pearson’s correlation analysis. We included two clinical variables in this study: the 17-item Hamilton Depression Rating Scale (HAMD), and 14-item Hamilton Anxiety Rating Scale (HAMA). There were no significant associations between clinical variables and regional MSN values of each region where MSN increased/decreased significantly in individuals with MDD compared to HC after FDR correction (Supplemental Result 10 and Table S5). An exploratory correlation analysis was also performed across all D–K 308 regions. We found that dorsal lateral prefrontal cortex exhibited a significant negative correlation with HAMD scores, whereas occipital cortices, middle/posterior cingulate cortex, and precentral cortex had positive correlations with HAMD scores (Fig. S9a). For HAMA scores, right dorsal lateral prefrontal cortex was negatively correlated, whereas left visual cortex and right temporal cortex were positively correlated (Fig. S9b).

### Cortical gene expression related to regional changes in MSN

**Fig. 3:**
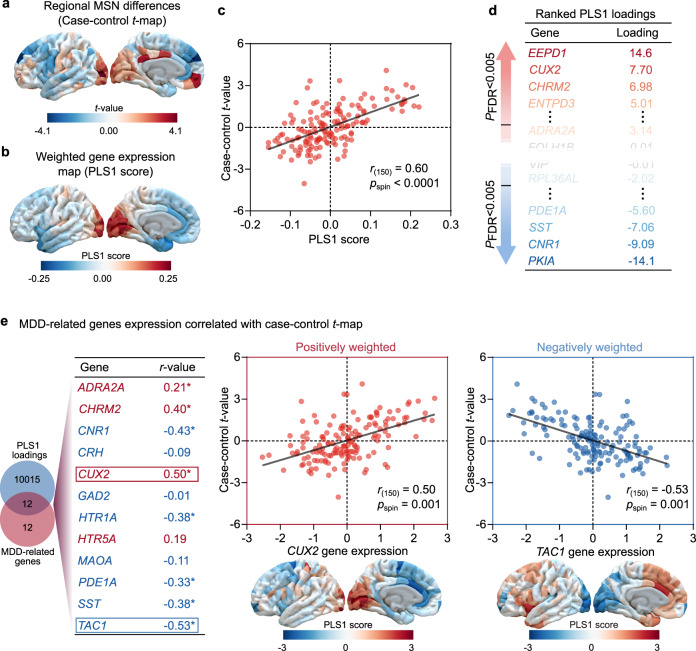
Gene expression profiles related to morphometric similarity differences. **a** Changes in regional morphometric similarity network (MSN) in the left hemisphere (unthresholded). **b** A weighted gene expression map of regional PLS1 scores in the left hemisphere (unthresholded). **c** A scatterplot of regional PLS1 scores (a weighted sum of 10,027 gene expression scores) and regional changes in MSN (Pearson’s *r*_(150)_ = 0.60, *p*_spin_ = 0.00). *p* value was not corrected for multiple comparisons, and was determined based on a one-sided test. **d** Ranked PLS1 loadings. **e** MDD-related genes from in situ hybridization in the adult human brain positively (i.e., *ADRA2A*: Pearson’s *r*_(150)_ = 0.21, adjusted *p*_spin_ = 0.04; *CHRM2*: Pearson’s *r*_(150)_ = 0.40, adjusted *p*_spin_ = 0.003; *CUX2*: Pearson’s *r*_(150)_ = 0.50, adjusted *p*_spin_ = 0.001; *HTR5A*: Pearson’s *r*_(150)_ = 0.19, adjusted *p*_spin_ = 0.14) and negatively (i.e., *CNR1*: Pearson’s *r*_(150)_ = −0.43, adjusted *p*_spin_ = 0.007; *CRH*: Pearson’s *r*_(150)_ = −0.09, adjusted *p*_spin_ = 0.28; *GAD2*: Pearson’s *r*_(150)_ = −0.01, adjusted *p*_spin_ = 0.43; *HTR1A*: Pearson’s *r*_(150)_ = −0.38, adjusted *p*_spin_ = 0.01; *MAOA*: Pearson’s *r*_(150)_ = −0.11, adjusted *p*_spin_ = 0.25; *PDE1A*: Pearson’s *r*_(150)_ = −0.33, adjusted *p*_spin_ = 0.03; *SST*: Pearson’s *r*_(150)_ = −0.38, adjusted *p*_spin_ = 0.04; *TAC1*: Pearson’s *r*_(150)_ = −0.53, adjusted *p*_spin_ = 0.001) correlated with regional changes in MSN. All *p* values were derived from spin tests and adjusted by FDR, and were determined based on one-sided tests. The asterisk represents *p* values that survived after FDR-corrected with *p* < 0.05.

We used the AHBA (http://human.brain-map.org)^37,38^, a whole-brain transcriptomic dataset, to obtain brain gene expressions (Supplemental Result 11.1). Because the AHBA dataset includes two right hemisphere data points alone (Table S6), only the left hemisphere was considered in our analysis^39^. As a result, a matrix (152 regions × 10,027 gene expression levels) of transcriptional level values was obtained (Supplemental Result 11.2). We then used partial least squares (PLS) regression^40^ to determine differences between regional MSN in the left hemisphere (Fig. 3a) and gene expressions (10,027 genes). The first component (PLS1) is defined as the spatial map that captures the greatest fraction of total gene expression variance across cortical areas. In the discovery cohort, PLS1 explained 36% of the variance (*p*_spin_ < 0.0001, this permutation test randomly “spins” the MSN map to account for spatial correlation). The distribution of the PLS1 weighted map reflects an anterior-posterior gradient of gene expression (Fig. 3b), which is interpreted as an areal variation in the transcriptional architecture of human cortex that is also captured in the MDD-related changes in the MSN map^41^. Notably, we found that the PLS1 weighted gene expression map was spatially correlated with the case-control *t*-map (Pearson’s *r*_(150)_ = 0.60, *p*_spin_ < 0.0001; Fig. 3c). We ranked the normalized weights of PLS1 based on univariate one-sample *Z* tests. We found 1747 PLS1+ (*Z* > 5) and 1237 PLS1− (*Z* < − 5) (all *p*_FDR_ < 0.005; Fig. 3d) positively (or negatively) weighted gene expressions were overexpressed (or under-expressed) as increased (or decreased) regional changes in MSN, respectively. In total, 2984 genes constituted the regional change in MSN gene list in individuals with MDD.

To further determine relationships between prior MDD-related gene expressions and regional changes in MSN, we first identified 24 MDD-related genes by searching with the disease term “depression” in the Category of the “Gene List” documentation from in situ hybridization data in the AHBA (help.brain-map.org/display/humanbrain/Documentation)^42^. Of these, we then selected the 12 genes that overlapped with the 10,027 background genes. Eight of twelve MDD-related genes exhibited significant correlations with regional changes in MSN (all *p*_FDR_ < 0.05; Fig. 3e), including five negative correlations (i.e., *CNR1*, *HTR1A*, *PDE1A*, *SST*, and *TAC1*) and three positive correlations (i.e., *ARRA2A*, *CHRM2*, and *CUX2*).

### Transcriptional correlates of MDD-related changes in MSN capturing patterns of gene upregulation

**Fig. 4:**
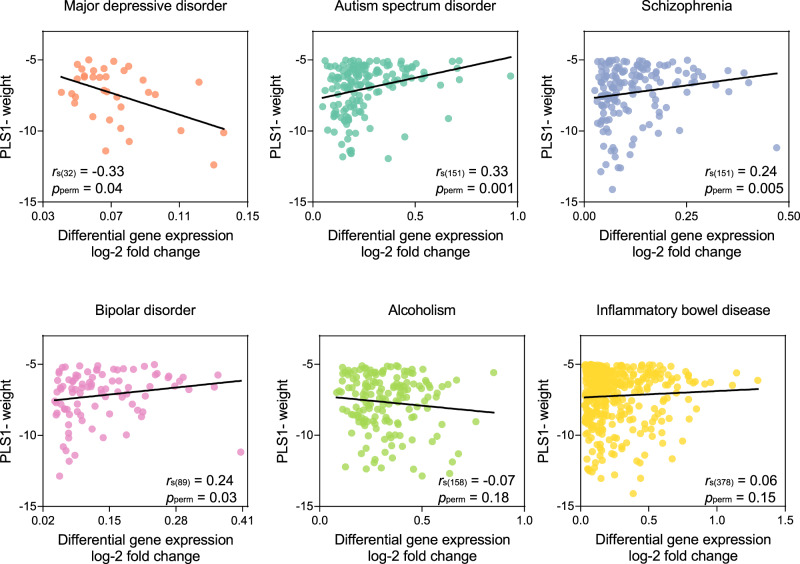
PLS1− weighted gene expressions of MDD-related changes in MSN associated with histological measures of differential gene expression of MDD and other disorders. The PLS1− weighted gene expression was associated with upregulated differential gene expression (DGE) postmortem in MDD reported by Gandal et al. (Spearman’s *r*_s(32)_ = −0.33, adjusted *p*_perm_ = 0.04, FDR-corrected). Associations between PLS1− weights and DGE were also evaluated for other brain disorders: autism spectrum disorder (Spearman’s *r*_s(151)_ = 0.33, adjusted *p*_perm_ = 0.001, FDR-corrected), schizophrenia (Spearman’s *r*_s(151)_ = 0.24, adjusted *p*_perm_ = 0.005, FDR-corrected), bipolar disorder (Spearman’s *r*_s(89)_ = 0.24, adjusted *p*_perm_ = 0.03, FDR-corrected), alcoholism (Spearman’s *r*_s(158)_ = −0.07, adjusted *p*_perm_ = 0.18, FDR-corrected), and inflammatory bowel disease (Spearman’s *r*_s(378)_ = 0.06, adjusted *p*_perm_ = 0.15, FDR-corrected). All *p* values were derived from permutation tests adjusted by FDR, and were determined based on one-sided tests.

We found that 34 genes overlapped between the PLS1− gene list (*Z* < − 5) and the genes that were significantly differentially expressed in postmortem brain tissue measurements of messenger RNA from case-control studies of MDD reported by Gandal et al. as upregulated^43^. Because of outliers in the gene set that can potentially inflate or deflate associated correlations^44^, we performed a Spearman’s correlation analysis to explore the associations. The PLS1− gene weights were correlated with MDD-related DGE values reported by Gandal et al.^43^: Spearman’s *r*_s(32)_ =−0.33, adjusted *p*_perm_ = 0.04, FDR-corrected (Fig. 4). Suggesting a degree of specificity across diagnostic groups, this negative relationship was particular to data from individuals with MDD and was not present in the other five disorders described in Gandal et al.^43^: schizophrenia (SCZ, *r*_s(151)_ = 0.24, adjusted *p*_perm_ = 0.005, FDR-corrected), bipolar disorder (BD, *r*_s(89)_ = 0.24, adjusted *p*_perm_ =0.03, FDR-corrected), autism spectrum disorder (ASD, *r*_s(151)_ = 0.33, adjusted *p*_perm_ = 0.001, FDR-corrected), alcoholism (*r*_s(158)_ = −0.07, adjusted *p*_perm_ = 0.18, FDR-corrected), and inflammatory bowel disease (IBD, *r*_s(378)_ = 0.06, adjusted *p*_perm_ = 0.15, FDR-corrected). Correlations for each disorder were calculated using genes that were common across significantly upregulated postmortem and PLS1− gene datasets. Of note, and similar to a previous study^32^, postmortem data from common psychiatric disorders including SCZ, BD, and ASD, showed effects in the opposite direction, indicating that the upregulated genes in these psychiatric disorders have higher gene expression than in MDD-related changes in MSN. There was no significant correlation between PSL1− gene weights and DGE values of downregulated genes in MDD.

### Enrichment pathways associated with changes in MSN

**Fig. 5:**
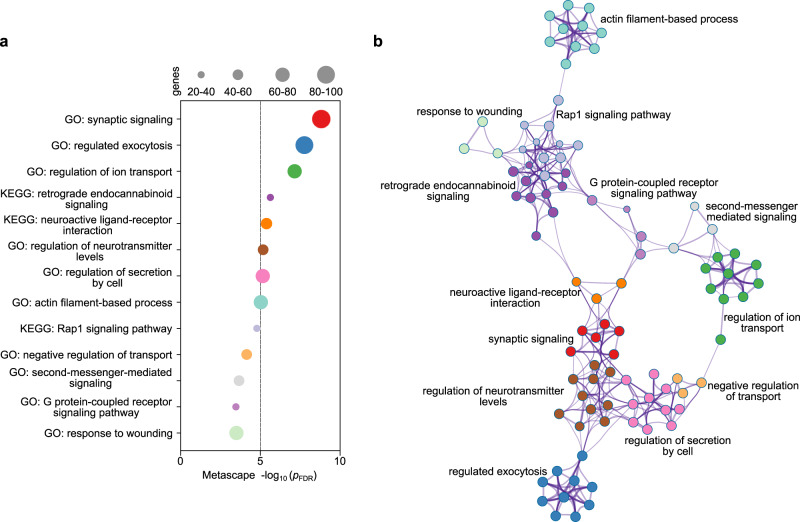
Functional enrichment of gene transcripts. **a** Ontology terms for PLS1− genes (*Z* < − 5, *p*_FDR_ < 0.05). The size of the circle represents the number of genes involved in a given term. **b** Metascape enrichment network visualization showing the intra-cluster and inter-cluster similarities of enriched terms. Each term is represented by a circle node, where its size is proportional to the number of input genes included in that term, and its color represents its cluster identity (i.e., nodes of the same color belong to the same cluster).

We aligned the gene ontology (GO) biological processes and Kyoto Encyclopedia of Genes and Genomes (KEGG) pathways with the PLS1− gene list using Metascape^45^. After correcting for enrichment terms (*p*_FDR_ < 0.05) and discarding discrete enrichment clusters, there were top ten significant GO biological processes, such as “synaptic signaling”, “regulated exocytosis”, and “regulation of ion transport”, and three KEGG pathways, including “retrograde endocannabinoid signaling”, “neuroactive ligand-receptor interaction”, and “Rap1 signaling pathway” (Fig. 5). The PLS1+ genes were enriched for GO biological processes (Supplemental Result 11.3), such as “signal release”, and “synaptic vesicle priming”, but not for KEGG pathways (Fig. S10 and Table S7). Genes that were downregulated postmortem in individuals with MDD were not correlated with weighted gene expressions of PLS1+.

Using recent genome-wide meta-analysis studies (GWAS)^46,47^, we examined whether there are shared enrichment pathways between polygenic risk for MDD and the PLS1− gene list. We performed a multi-gene-list meta-analysis^45^ between the PLS1− gene list and genes that were significantly associated with the MDD phenotypes. We found that enrichment pathways of the PLS1− gene list contained six of seven pathways of genes from GWAS studies. The enrichment pathways included “cognition”, “Ras protein signal transduction”, “regulation of ion transport”, “synaptic signaling”, “synapse organization”, and “cell–cell adhesion via plasma membrane adhesion molecules” (Supplemental Result 11.4 and Fig. S11). These results indicate that functional roles of PLS1− genes are not only consistent with previous studies, but also provide additional complementary functional information.

### Transcriptional signatures for canonical cell types

**Fig. 6:**
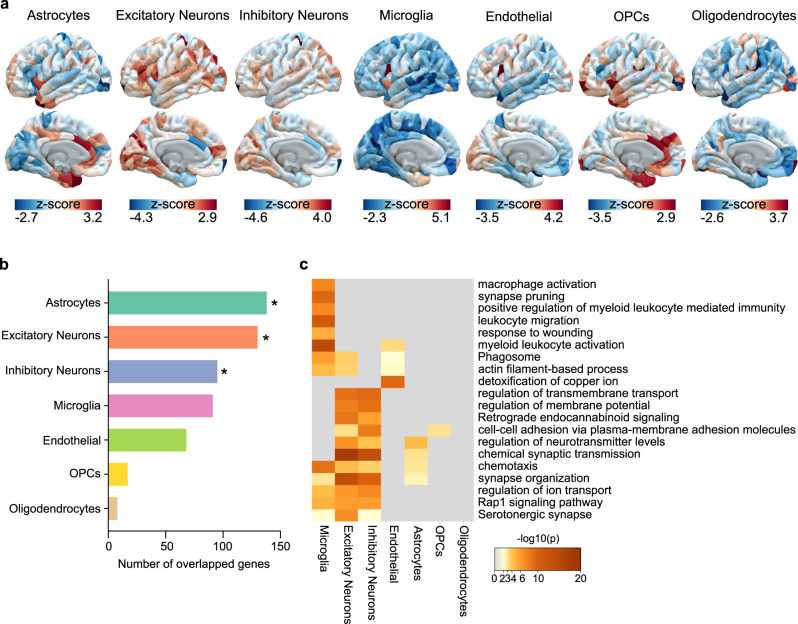
Cell type-specific expression to changes in MSN-related genes. **a** Regional gene expression maps of each cell type from overlapping genes between PLS1− gene list and each cell type-specific genes. **b** The number of overlapping genes for each cell type (Astrocytes: number = 138, adjusted *p*_perm_ = 0.001; Excitatory neurons: number = 130, adjusted *p*_perm_ = 0.004; Inhibitory neurons number = 95, adjusted *p*_perm_ = 0.04; Microglia: number = 91, adjusted *p*_perm_ = 0.32; Endothelial: number = 68, adjusted *p*_perm_ = 1.00; Oligodendrocyte precursors (OPCs): number = 17, adjusted *p*_perm_ = 0.32; Oligodendrocytes: number = 8, adjusted *p*_perm_ = 1.00). All permutated *p*_perm_ values were adjusted by FDR, and were determined based on one-sided tests. An asterisk represents *p* values that survived after FDR-corrected with *p* < 0.05. **c** Gene ontology terms enriched for changes in MSN-related genes for the cell types.

To further refine our analysis, and considering cellular diversity in the brain, we took an indirect approach to assign PLS1− genes to seven canonical cell classes^19^: excitatory neurons, inhibitory neurons, microglia, endothelial cells, oligodendrocytes, astrocytes, and oligodendrocyte precursors (OPCs), identifying specific cell types enriched for MSN alterations in our analysis. We first visualized the distribution of gene expression in each cell type (Fig. 6a). A number of genes in the PLS1− gene list was significantly involved in astrocytes (number = 138, adjusted *p*_perm_ = 0.001, FDR-corrected), excitatory neurons (number = 130, adjusted *p*_perm_ = 0.004, FDR-corrected), and inhibitory neurons (number = 95, adjusted *p*_perm_ = 0.04, FDR-corrected) (Fig. 6b). Notably, consistent with previous single-cell sequencing in MDD, we found that the cell type of gene expression showed a similar cell type of excitatory neurons^48^. Confirming our strategy, enrichment analysis using cell type-specific genes revealed that changes in MSN in individuals with MDD were significantly enriched for biological processes associated with inflammation in microglial and neuronal cells (Fig. 6c). Changes in MSN identified in neuronal cells were enriched for GO terms including “serotonergic synapse”, “synapse organization”, and “chemical synaptic transmission”. Together, our approach identified changes in MSN-related gene expression to unique cell types, allowing us to pinpoint specific cell types known to be associated with MDD pathology.

### Reproducibility of MDD-related changes in MSN and transcriptomic profile

We validated the MDD-related pattern of changes in MSN and MDD-related changes in MSN linked to transcriptional profiles in an independent replication cohort. There was no difference in image quality, age, and sex between individuals with MDD and HC in the replication cohort (Supplemental Result 1 and Fig. S1). The identically derived MDD-HC (case-control) *t*-map from the replication cohort was spatially concordant to the discovery cohort (Pearson’s *r*_(306)_ = 0.43, *p*_spin_ = 0.0002; Fig. S12a, b). This moderate, but significant relationship may be due to the limited size of replication cohort. In addition, not all *t*-maps of changes in MSN yielded similar sized correlations (Supplemental Result 12.1 and Fig. S12c).

**Fig. 7:**
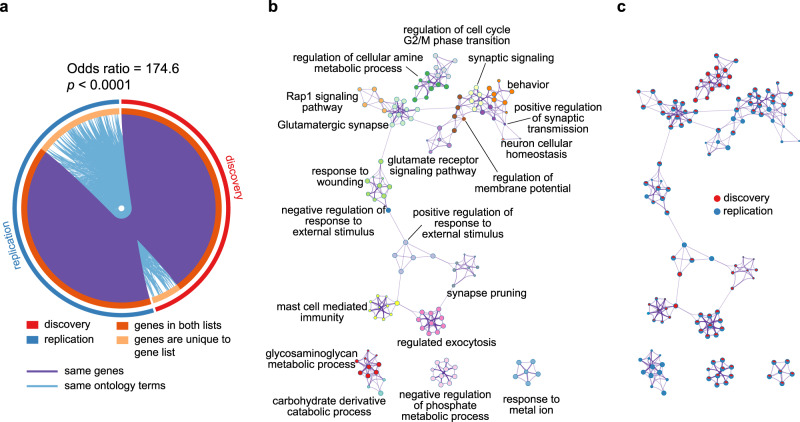
Validation of transcriptional enrichments of changes in MSN. **a** Circos plot of genes overlapped between discovery and replication cohorts. **b** A subset of representative terms from all clusters. **c** The same enrichment network with its nodes displayed as pie sections. Each pie sector is proportional to the number of hits originating from a gene list.

To further investigate the validation of transcriptional enrichments of changes in MSN, a multi-gene-list meta-analysis^45^ of PLS1− was also performed between discovery and replication cohorts. In the replication cohort, we found that 2150 PLS1+ (*Z* > 5) genes, and 1503 PLS− (*Z* < − 5) genes were significantly overexpressed in cortical regions, consisting of 3653 regional MSN gene list differences. The gene lists in the discovery and replication cohorts were highly overlapped: odds ratio (OR) = 109.5, *p* < 0.0001. In addition, we identified the overlapped enrichment pathways between discovery and replication cohorts where there was a significant overlap of PLS1− genes: OR = 174.6, *p* < 0.0001 (Fig. 7a). After correcting for enrichment pathways, several ontological terms survived (Fig. 7b), which were the same as those from the discovery enrichment analyses, including “synaptic signaling”, and “Rap1 signaling pathway”. The overlapping ontology terms between discovery and replication cohorts concentrated on “synaptic signaling”, “Glutamatergic synapse”, “Rap1 signaling pathway”, “behavior”, “regulated exocytosis”, “negative regulation of phosphate metabolic process”, and “response to metal ion” (Fig. 7c). For visualization, uncorrected overlapping ontology terms are shown in Fig. S13 (Supplemental Result 12.2). Significantly overlapping ontology terms support the generalized relationship between gene expression and the MDD-related changes in MSN.

## Discussion

Using structural MRI to define replicable maps of individuals with MDD-related differences in anatomical organization, we found that this cortical pattern of MDD effects was significantly associated with normative gene expression gradients enriched for MDD-related genes. Specifically, the changes in MSN-related gene transcripts (PLS1− ): (i) were enriched for prior defined MDD-related genes, and exhibited almost the same ontological terms as those genes identified from GWAS studies; (ii) were associated with genes that were significantly upregulated in prior postmortem material from MDD; and (iii) were ontologically enriched for synapse-related terms that were validated in a replication cohort. In addition, we mapped MDD-related genes to biological processes associated with microglial and neuronal cells. These findings reveal MSN phenotypes in MDD, and bridge the gap between transcriptome and neuroimaging promoting an integrative understanding of MDD.

### MDD-related changes in MSN

Rather than using single anatomical and morphometric features, such as cortical thickness, curvature, and volume, MSNs combine information across multiple cortical features in a single participant^14,17–19^. DWI indirectly characterizes myeloarchitecture of brain regions while T1w assesses macroscopic morphology of the cortex. According to previous studies^12^, high regional MSN represents cytoarchitectonically similar networks that may be more likely to be axonally connected to each other. However, this macroscale brain organization cannot be evaluated with the limited spatial resolution and cellular specificity of T1w MRI and current connectomic analysis techniques (i.e., DWI-based tractography and structural covariance analysis)^49^. Nevertheless, in this study we have interpreted regions with altered MSN in individuals with MDD as indicating that there may be corresponding architectonic similarity or architectonic differentiation between these regions and the rest of the cortex^18^. MDD shares common brain alterations with other psychiatric and neurological disorders^49,50^, as well as having diagnosis-specific features. Transdiagnostic patterns of gray matter loss are located in the anterior insula and dorsal anterior cingulate cortex^51^; whereas transdiagnostic patterns of anatomical connectome are related to highly-connected hubs^52^. However, diagnosis-specific effects volumetric changes are found only in MDD and SCZ^51^. Our reliable MSN alterations showed a general convergence of affected regions with other psychiatric disorders in regions including the medial prefrontal cortex, and isthmus cingulate.

The MDD-related changes in MSN appear to accumulate in specific systems: the functionally defined visual network and the cytoarchitectonically defined dysgranular secondary sensory class. Both of these systems include visual areas. However, note the differences in the anatomical distributions between the “Yeo visual” and “von Economo secondary sensory” systems; results with the Yeo visual network are focused on the lower and higher visual areas, whereas with the von Economo secondary sensory class they are mainly focused on the lower visual areas and orbitofrontal cortices. The von Economo atlas has additional heuristic value because it could be used to explore the cellular underpinnings of MDD^53^.

### MDD-risk genes related to changes in MSN

MDD-related changes in MSN may be due to a host of factors such as genetic, molecular, and neuronal alterations. Recently, human imaging genetics has emerged as a powerful strategy for understanding the molecular basis of brain connectome organization^18,22,29,30^. Using the multivariate PLS method, we found cortical patterns of weighted gene expression that were significantly co-localized with changes in MSN, and further identified significantly weighted genes in the first PLS component that may have roles in the pathogenesis of MDD.

MDD-related gene analysis suggested that a substantial part (8/12) was related to changes in MSN. The discovered gene *SST* codes for a neuromodulatory peptide expressed in a subtype of GABA neurons that inhibits the dendritic compartment of excitatory pyramidal neurons^54^. Reduced *SST* gene expression has frequently been observed postmortem in brains of individuals with MDD^55,56^. *SST* was the third strongest negatively correlated gene, with *TAC1* showing a stronger inverse association, which is a gene earlier noted to be involved in MDD and related to depression-like behaviors^57–60^. Similar to *SST*, *TAC1* is also a gene related to neuron excitation and behavioral responses^60^. The underlying pathogenetic mechanism of both positively versus negatively correlated genes presently remains unclear. A potential explanation may lie in the distinct types of cortical interneurons between genes marked by neuropeptides (e.g., *SST* and *TAC1*) and genes related to biological processes, such as genes involved in protein coding^42^.

The PLS1− gene list was specifically associated with genes that were significantly upregulated in postmortem individuals with MDD. Large-scale GWAS has identified the shared significant genetic commonalities across major psychiatric disorders^61,62^, and MDD shows positive genetic correlations with most other psychiatric disorders^62,63^. Consistent with potential disorder-specific associations, negatively weighted gene expression profiles in this study showed positive correlations with genes differentially expressed in postmortem case-control studies of SCZ, BD, and ASD suggestive of potential converging pathophysiological mechanisms in these common psychiatric disorders^61^, which will require future studies to validate this proposition in the context of neuroimaging-transcriptomics. Additionally, there were significantly more upregulated genes in the list of negatively weighted genes with changes in MSN, indicating that genes with increased brain postmortem transcription in MDD were overexpressed in cortical areas with lower levels of changes in MSN. An important future direction involves quantifying the degree to which genetic influences of risk for MDD may be directly mediated by their effects on MSN.

### Weighted gene expressions enriched for functional annotations

PLS1− identified a gene expression profile with high expression in the frontal and temporal cortices. The subset of 1237 negatively weighted genes comprised a dense, topologically clustered interaction network that was enriched for several GO biological processes and KEGG pathways. The highly overlapping PLS1− genes were associated with the same ontological terms in both discovery and replication cohorts, suggesting a generalization of transcriptional signatures of changes in MSN.

The identified KEGG pathways (i.e., “retrograde endocannabinoid signaling” and “neuroactive ligand-receptor interaction”) have been reported to modulate a wide variety of synaptic neurotransmissions or neural functions, including cognition, motor control, and pain^64,65^. Abnormalities or dysregulations of these pathways have been implicated in MDD^66^. Moreover, the endocannabinoid signaling system is a potential antidepressant candidate^67^ as it may help reverse the acute and chronic stress response, and produce antidepressant physiological changes. The endocannabinoid signaling system deserves additional study as a potential target for therapeutic intervention^64,68^.

Our identified GO biological processes were related to responses to stimuli and synaptic transmission, indicating that some negatively weighted genes had diverse molecular functions^65^. In particular, the discovered pathway “synaptic signaling”, which influences synaptic maturation and stability^69^, was one of the replicable pathways between discovery and replication cohorts, showing a high Metascape value out of all other pathways. Loss of synapses has been reported to produce depressive behavior in rodent models^70^. The cluster of interactive proteins related to “G protein-coupled receptors” (GPCRs) signaling pathways mediate most cellular responses to hormones and neurotransmitters^71^. As suggested for the “synaptic signaling” pathway, GPCRs signaling pathways are implicated in the pathophysiology and pharmacology of MDD. These findings highlight GPCRs as potential therapeutic targets for MDD, which warrant follow-up analyses.

For validation, an additional enrichment analysis helped us specify the gene ranks related to changes in MSN. Consistent with GWAS in MDD, the same ontology terms, especially synapse-related terms, support the reliability and sensitivity of genes identified by PLS in this study^46,47^. In addition, the multi-gene-list result exhibits several other enrichment pathways which are found in genes related to changes in MSN, but not in genes of GWAS, and thus the genes obtained by PLS might provide additional enrichment information for MDD.

### Cellular characterization of the MDD-related genes

We showed that cellular organization of the human brain provides a biological mechanism that can translate genes of MDD-related brain alterations into MDD-related alterations of specific cell types. The density and form of cell abnormalities (in astrocytes, microglia, or oligodendrocytes) plays an important role in psychiatric disorders, including ASD, BD, MDD, and SCZ^63^. Alterations in cortical thickness for major psychiatric disorders have been related to gene expression specific to astrocytes (except for BD) and microglia (except for obsessive-compulsive disorder)^63^. Astrocytes were the greatest proportion in gene ranks obtained by PLS on changes in MSN in MDD, and have also been considered as a promising target for mood disorder interventions^72^. The dysfunction of astrocytes influences synaptic activity, and astrocytes can modulate neuronal circuits and behavior. Similarly, astrocytes were found to be consistent cell associates of depression using different neuroimaging measurements^32^. Furthermore, leveraging multiple morphometric features, we identified that the most enriched pathway was related to microglia, aligning with prior reports^73^. Microglia play crucial roles in the regulation of ongoing structural and functional processes, from individual synapses to neural circuits and behavior^74^. The disturbances of microglia activation could influence immune functioning of the brain, synaptic plasticity and mood under physiologically strained conditions. Finally, we found the dysregulation of gene expression in MDD was related to excitatory and inhibitory neurons, which was consistent with the single-nucleus transcriptomics study in MDD^48^. In recent years, the target cell types in MDD pathophysiology have expanded from excitatory neurons to inhibitory interneurons^75^. The identified MDD-related cell types verified the validity of the gene ranks obtained from changes in MSN and enabled us to explore the biology of human disorders using data from postmortem human brain tissue.

### Methodological considerations and future directions

Several methodological issues have to be considered. First, considering the MSN as a combination of multiple morphometric features rather than a functional index, we used the D–K atlas to anatomically parcellate the human cerebral cortex^14,18,19^. The atlas is useful for both morphometric and functional studies of the cerebral cortex^76^ making it likely that this atlas encompasses multiple functional territories. Second, we considered connection strength in MSN in line with previous studies^18,19^. It could be meaningful to link MSN topological properties to transcriptomic and cellular correlates of MDD in future work. Third, this study collected limited clinical variables related to MDD symptoms. Future studies should include a more comprehensive set of metrics to characterize the clinical significance of MDD-related differences. Similarly, given the evidence for an effect of body mass index (BMI) on brain structure^77^, future studies should include BMI as an important factor in their MSN analyses. Fourth, the remarkable public AHBA gene data were measured postmortem in six participants without psychiatric diagnoses, which limited examination of transcriptome–neuroimaging associations across groups, and possibly places individual effects out of scope. In addition, the AHBA only included data for the right hemisphere for two participants. Thus, the relationship between genes and MDD-related changes in MSN does not represent the condition of the entire brain. Finally, although the identified cell types in this study have been used as biomarkers for potential targets for therapeutic intervention, future work should employ longitudinal data (e.g., pre-treatment vs. post-treatment) to validate the findings.

This study linked MSN phenotypes to gene expression levels, supporting the idea that synapse-related terms are implicated in the pathophysiology and pharmacology of MDD. We further showed that abnormalities of astrocytes, microglial, and neuronal cells may link to MDD-related changes in MSN, which might denote future treatments. Crucially, despite not requiring access to any postmortem brain tissue from patients, we can screen the MSN-related differences brain-wide gene expression and cell types to capture molecularly validated anatomical differences in psychiatric patients.

## Methods

### Participants

The discovery cohort included individuals with MDD (*n* = 242) and age- and sex-matched HC (*n* = 231). Individuals were recruited from the First Affiliated Hospital of Chongqing Medical University. The replication cohort included individuals with MDD (*n* = 51) and age- and sex-matched HC (*n* = 43), and individuals were acquired and scanned from the First Affiliated Hospital of Anhui Medical University. Individuals with MDD were diagnosed as having current depressive status using the Structural Clinical Interview for Diagnostic and Statistical Manual of Mental Disorders-IV^78^ by experienced psychiatric physicians. Depression severity was assessed by the 17-item HAMD. Anxiety severity was assessed by 14-item HAMA. Individuals with MDD were excluded if they: (i) were <18 years or >65 years; (ii) had HAMD <8; (iii) had major neurological or other psychiatric disorders; and (iv) had MRI abnormalities, or had any metal or electronic implants. HC were recruited with the following eligibility criteria: (i) no other axis I psychiatric disorders or neurological disorders, (ii) no axis II antisocial or borderline personality disorders, and (iii) no history of psychiatric illness among their first-degree relatives.

The discovery study was approved by the Ethics Committee of Southwest University and First Affiliated Hospital of Chongqing Medical University. The replication study was approved by the Medical Ethics Committee of Anhui Medical University. All study protocols were performed according to the Helsinki Declaration of 1975. All participants provided informed consent and understood the purpose, benefits, and potential risks to participate in this study.

### Multi-neuroimaging data acquisition

#### Discovery cohort

The T1w and DWI images were collected using a Siemens Trio 3.0 T scanner (Siemens Medical, Erlangen, Germany) at the Southwest University Center for Brain imaging. The 3D T1w images were acquired as follows: repetition time (TR) = 1900 ms, echo time (TE) = 2.52 ms, flip angle = 9°, field of view (FOV) = 256 × 256 mm^2^, matrix size = 256 × 256, voxel size = 1 × 1 × 1 mm^3^, and slices = 176. Subsequently, the DWI images were acquired using a diffusion-weighted, single shot, spin-echo, gradient-echo planar imaging sequence as follows: TR = 11,000 ms, TE = 98 ms, FOV = 256 × 256 mm^2^, matrix size = 128 × 128, voxel size = 2 × 2 × 2 mm^3^, slices = 60, one volume without diffusion weighting *b* = 0 s/mm^2^, 30 non-collinear directions *b* = 1000 s/mm^2^. To improve the signal to noise ratio, the entire sequence was repeated three times.

#### Replication cohort

The structural and DWI images were collected using a GE 3.0 T scanner (Discovery 750; GE Healthcare, Milwaukee, WI) at the University of Science and Technology of China. The 3D T1w images were acquired as follows: TR = 8.16 ms, TE = 3.18 ms, flip angle = 12°, FOV = 256 × 256 mm^2^, matrix size = 256 × 256, voxel size = 1 × 1 × 1 mm^3^, and slices = 188. Subsequently, the DWI data for each subject were acquired using a diffusion-weighted, single shot, spin-echo, gradient-echo planar imaging sequence as follows: TR = 6900 ms, TE = 60.4 ms, FOV = 256 × 256 mm^2^, matrix size = 128 × 128, voxel size = 2 × 2 × 2 mm^3^, slices = 69, five volumes without diffusion weighting *b* = 0 s/mm^2^, 64 non-collinear directions *b* = 1000 s/mm^2^.

### Data preprocessing

The three-dimensional T1w images were preprocessed in surface-based space using FreeSurfer (v6.0, http://surfer.nmr.mgh.harvard.edu/). Briefly, the cortical surface was reconstructed using skull stripping, segmentation of brain tissue, separation of hemispheres and subcortical structures, and construction of the gray/white interfaces and the pial surfaces^14^. The DWI images were preprocessed on volumetric space using FMRIB Software Library (FSL, v6.0, https://fsl.fmrib.ox.ac.uk/fsl/fslwiki). Briefly, the DWI images were corrected for the eddy-current-induced distortions and head movements. Diffusion tensor models were then estimated using linear least squares fitting.

Participants were excluded if they had images with poor scan quality (Supplemental Result 1). To further check for differences in motion and image quality between groups, the Euler number was calculated for each T1w image^79^.

### Construction of MSN

The cortical surfaces were divided into 308 spatially contiguous regions^14,18,80^ derived from the 68 cortical regions in the D–K atlas^76^. This parcellation produced approximately equal size (~500 mm^2^) for each region, using a backtracking algorithm^80^, which minimizes the influence of the variability in parcel sizes^14,18,19,81^. This parcellated D–K atlas was transformed to each participant’s surface to obtain an individual surface parcellation which was then interpolated and expanded to the participant’s DWI volumes^14,18^. For each region, seven features from the T1w and DWI images were extracted^18^, including surface area, cortical thickness, gray matter volume, Gaussian curvature, mean curvature, fractional anisotropy, and mean diffusivity. For each participant, each morphometric feature vector was *z*-normalized across regions to account for variation in value distributions between the features^14,18^. Pearson’s correlation analysis was then performed on the morphometric feature vector between each paired cortical region, forming a 308 × 308 MSN (no thresholding) for each participant. Additional connection densities (10‒90% in 10% increments) were used to validate the thresholding effect on construction of MSN^14^.

### Case-control analysis of the MSN

The regional MSN was calculated by using the sum of weighted correlation coefficients between a given region and its correlations to all other regions. To estimate the spatial pattern, regional MSN was averaged across all HC participants. To examine the case-control differences, a LRM was conducted with regional MSN values as the dependent variable. Age, sex, and education level were added as covariates. This model was fitted for each region, and the two-sided *t*-statistic (contrast = MDD ‒ HC) was extracted. For case-control comparisons in MSN values of each region (MSN_i_), the following model was used: MSN_i_ = intercept + β_1_ × (age) + β_2_ × (sex) + β_3_ × (education). Although there was no difference of TIV between individuals with MDD and HC, we also reanalyzed the case-control differences including TIV as a covariate in the LRM. Significance was set at *p* < 0.05 with FDR correction for multiple comparisons across 308 regions to control type I error.

### Estimation of regional gene expressions

The AHBA dataset (http://human.brain-map.org) bridges the gap between regional changes in MSN and transcriptomes^37^. Brain-wide gene expressions were measured in six postmortem brains (age = 42.50 ± 13.38 years; male/female = 5/1) with 3702 spatially distinct samples (Supplemental Result 11.1 and Table S6). The AHBA dataset was processed according to Arnatkevic et al.^39^. The six steps of preprocessing were as follows: (i) verifying probe-to-gene annotations using the Re-annotator toolkit^82^; (ii) filtering of probes (intensity-based filtering) that do not exceed background noise, excluding at least 50% of all samples across participants; (iii) probe selection, selecting the highest correlation to RNA-seq data; (iv) samples assignment to the D–K 308 atlas within 2 mm Euclidean distance of a parcel; (v) normalization of expression measures using a scaled robust sigmoid for each participant; and (vi) gene set filtering based on differential stability (Supplemental Result 11.2). Because the AHBA dataset included only two right hemisphere data, only the left hemisphere was considered in our analysis^39^. Thus, a mean of all samples in a region was calculated to obtain the matrix (152 regions × 10,027 gene expression levels) of transcriptional level values.

### Regional changes in MSN and gene expression

PLS regression^40^ was used to determine the relationship between regional changes in MSN (*t*-values from 152 cortical regions in the left hemisphere) and transcriptional activity for all 10,027 genes. Gene expression data were used as predictor variables of regional changes in MSN in the PLS regression. The first component of the PLS (PLS1) was the linear combination of gene expression values that was most strongly correlated with regional changes in MSN. Permutation testing based on spherical rotations, to account for spatial autocorrelation, of the MSN map (5000 times)^33^ was used to test the null hypothesis that PLS1 explained no more covariance between the MSN map and whole-genome expression than expected by chance^29^. Bootstrapping was used to estimate the variability of each gene’s PLS1, and the ratio of the weight of each gene to its bootstrap standard error was used to calculate the *Z* scores and rank the genes according to their contributions to PLS1^18^. The set of genes with an FDR of 5‰, either positive, PLS1+ , or negative, PLS1− , was the regional changes in MSN gene list. This procedure was also conducted on the replication cohort.

### Analysis of MDD-related genes from in situ hybridization (ISH) gene expression

We selected prior defined MDD-related genes from the “Genes characterized by ISH in 1000 gene survey in cortex” from the AHBA (help.brain-map.org/display/humanbrain/Documentation) that integrates all available datasets. The disease-related genes identified were based on published literature as being relevant to depression (24 genes), and other diseases^42^: *ADRA2A*, *AVPR1B*, *CHRM2*, *CNR1*, *CREB1*, *CRH*, *CRHR1*, *CRHR2*, *CUX2*, *GAD2*, *GPR50*, *HTR1A*, *HTR1B*, *HTR1D*, *HTR3A*, *HTR5A*, *MAOA*, *PDE1A*, *SLC6A2*, *SLC6A4*, *SST*, *TAC1*, *TPH1*, and *TPH2*. These genes are known to be involved in physiological pathways implicated in the diseases.

To explore the contribution of the MDD-related genes in the PLS analysis, we first obtained the overlapped genes from the 24 MDD-related gene list and 10,027 background genes. Then we estimated the associations between overlapped gene expressions and case-control changes in MSN in the left hemisphere. Significance was set at *p* < 0.05 with FDR correction for multiple comparisons.

### Brain disorders’ differential expression analysis

We tested whether transcriptionally MDD-related dysregulated genes in postmortem brain tissue measurements of messenger RNA are expressed most in cortical regions that are morphometrically correlated to MDD. The MDD-related dysregulated gene list reported by Gandal et al.^43^ included 1992 upregulated and 2093 downregulated (*p* < 0.05) genes. Genes used for sequent correlation analyses were common across significantly upregulated or downregulated postmortem and PLS1− gene datasets. Spearman’s correlation analysis^44^ was used to determine relationships between PLS1− gene weights and DGE values of up or downregulated genes. The above-mentioned analysis was also applied for ASD, SCZ, BD, alcoholism, and IBD, with diagnoses from Gandal et al.^43^. Significance was set at *p* < 0.05 with FDR correction.

### Enrichment analysis

Metascape analysis (https://metascape.org/gp/index.html#/main/step1) provides automated meta-analysis tools to understand either common or unique pathways in 40 independent knowledge bases^45^. The PLS1+ (*Z* > 5) or PLS1− (*Z* < − 5) was input into the Metascape website, and the obtained enrichment pathways were thresholded for significance at 5%, corrected by the FDR.

We tested whether the PLS1− gene list shared enrichment pathways with polygenic risk for MDD from recent GWAS^46,47^. A multi-gene-list meta-analysis^45^ was performed to facilitate the understanding of pathways (and pathway clusters) that are shared between, or selectively ascribed to, specific gene lists. Routine comparative approaches include the use of Venn diagrams to identify hits that are common or unique to gene lists. However, when multiple gene lists are analysed, the identification of consistent underlying pathways or networks are more critical^45^, because previous studies have reported that an overlap between OMICs datasets is more readily apparent at the level of pathways or protein complexes^83,84^. Thus, the PLS1− gene list and genes from GWAS in individuals with MDD were submitted to the Metascape website to compare an arbitrary number of gene lists across both gene identities and ontologies. All obtained pathways were thresholded for significance at 5%, corrected by the FDR.

### Assigning MDD-related genes to cell types

To obtain gene sets from each cell type, we compiled data from five different single-cell studies using postmortem cortical samples of human postnatal participants. This approach avoids bias based on acquisition methodology, analysis, or thresholding, and led to the initial inclusion of 58 cell classes^19^, many of which were overlapping based on nomenclature and/or constituent genes. Following the procedure in Seidlitz et al.^19^, we organized cell types into seven canonical classes: microglia, endothelial cells, oligodendrocyte precursors, oligodendrocytes, astrocytes, excitatory, and inhibitory neurons. Two studies did not subdivide neurons into excitatory and inhibitory sets, and thus these gene sets were excluded from this cell-class assignment. Additionally, one study included the annotation of the “Per” (pericyte) type, and thus this gene set was excluded.

To assign MDD-related genes obtained by PLS analysis to cell types, we overlapped the gene set of each cell type with the PLS1− rank gene list. The *p* value of the number of overlapped genes in each cell type was obtained by a permutation test^29^, and corrected by FDR with *p* < 0.05. Then we calculated an average expression for each cell-class gene set in each of the 152 regions of the AHBA parcellation. To explore the enrichment pathways in genes involved in each cell type, we performed enrichment analysis. All obtained pathways were thresholded for significance at 5%, corrected by the FDR.

### Null models

The *p* values in this study were tested against two categories of null models. The first null model was based on the spin test. We used a “spin”-based method to correct for potential confounding effects of spatial autocorrelation (https://github.com/frantisekvasa/rotate_parcellation)^33^. The spin test is a spatial permutation method based on angular permutations of spherical projections at the cortical surface. Critically, the spin test preserves the spatial covariance structure of the data and as such is far more conservative than randomly shuffling locations, which destroys the spatial covariance structure and produces an unrealistically unconservative null distribution. Specifically, we first generated 5000 random spatial rotations (i.e., spins) of the cortical regions to generate a null distribution. Then, the *p*_spin_ values were obtained by comparison to the null models (<5th, or >95th percentile).

The second null model is based on a permutation test. In the “Transcriptional correlates of MDD-related changes in MSN capturing patterns of gene upregulation” section, we randomly relabeled the dysregulated sets^43^ 5000 times across all genes that were found to be differentially expressed to test the null hypothesis that the PLS1− weights were not related to the DGE of MDD-related genes other than by chance. A distribution of the test statistic was obtained. The *p*_perm_ were obtained by the occupied null models (<5th, or >95th centile), and corrected by FDR. In the “Transcriptional signatures for canonical cell types” section, we resampled the genes involved in cell types 5000 times to test the null hypothesis that the PLS1− gene list was randomly assigned to different cell types. The *p*_perm_ values were obtained from the null models (<5th, or >95th centile), and corrected by FDR with *p* < 0.05.

### Validation analysis

The above case-control changes in MSN were validated in the replication cohort. Leveraging the identical strategy used in discovery cohort, we obtained the case-control *t*-map in the replication cohort using LRM regressing out age, sex, and education level. To test the replicability of regional changes in MSN, a spatial similarity analysis was conducted on *t*-value maps between discovery and replication cohorts^18,85^.

For validating the MDD-related gene list obtained by MSN analysis, a multi-gene-list meta-analysis was conducted between the PLS1− gene lists of the discovery and replication cohorts. All obtained pathways were thresholded for significance at 5%, corrected by the FDR. The degree of overlapped genes was measured by the OR.

### Reporting Summary

Further information on research design is available in the Nature Research Reporting Summary linked to this article.

## Supplementary information

Supplementary Information

Description of Additional Supplementary Files

Supplementary Data 1

Reporting Summary

## Data Availability

Human gene expression data that support the findings of this study are available in the Allen Brain Atlas (“Complete normalized microarray datasets”, https://human.brain-map.org/static/download). MDD-related genes from ISH can be obtained from http://help.brain-map.org/download/attachments/2818165/HBA_ISH_GeneList.pdf?version=1&modificationDate=1348783035873&api=v2. Dysregulated genes in postmortem brain tissue measurements of messenger RNA is from the raw Gandal et al.^43^ dataset (https://science.sciencemag.org/highwire/filestream/705756/field_highwire_adjunct_files/1/aad6469_Gandal_SM_Data-Table-S1.xlsx). Compiled cell-specific gene set list from all available large-scale single-cell studies of the adult human cortex can be obtained from the raw Seidlitz et al.^19^ dataset (https://static-content.springer.com/esm/art%3A10.1038%2Fs41467-020-17051-5/MediaObjects/41467_2020_17051_MOESM8_ESM.xlsx). All data supporting the findings of this study are provided within the paper and its supplementary information. All additional information will be made available upon reasonable request to the authors. The PLS1‒ and PLS1+ gene lists and *Z*-score weights from the discovery cohort are provided in Supplementary Data 1.

## Code availability

The neuroimaging preprocessing software is freely available (FreeSurfer v6.0, http://surfer.nmr.mgh.harvard.edu/, and FSL v6.0, https://fsl.fmrib.ox.ac.uk/fsl/fslwiki). The codes for MSN analysis and PLS are openly available at https://github.com/SarahMorgan/Morphometric_Similarity_SZ. The code for gene expression analysis can be found at https://github.com/BMHLab/AHBAprocessing. The probe-to-gene annotations were obtained by the Re-annotator toolkit (v1.0.0, https://sourceforge.net/projects/reannotator/). Gene enrichments were analysed at https://metascape.org/gp/index.html#/main/step1. The code for spatial permutation testing can be found at https://github.com/frantisekvasa/rotate_parcellation. The brain maps were presented using Surf Ice (v1.0.20190902, https://www.nitrc.org/projects/surfice/).
