# Supplementary material for: Cortical structural differences in major depressive disorder correlate with cell type-specific transcriptional signatures
Source: Nat Commun. 2021 Mar 12;12:1647. doi: 10.1038/s41467-021-21943-5 (PMC7955076; doi:10.1038/s41467-021-21943-5)
Supplement: Supplementary file 1 — Supplementary Information [file 41467_2021_21943_MOESM1_ESM.pdf]

Supporting information for

Cortical structural differences in major depressive disorder correlate with cell type-specific transcriptional signatures

Running title: Transcriptional and Cellular Profiles in MDD

Jiao Li[1,2], Jakob Seidlitz[3,4], John Suckling[5], Feiyang Fan[1,2], Gong-Jun Ji[6], Yao Meng[1,2], Siqi Yang[1,2], Kai Wang[6], Jiang Qiu[7], Huafu Chen[1,2] ✉, Wei Liao[1,2] ✉

[1] The Clinical Hospital of Chengdu Brain Science Institute, School of Life Science and Technology, University of Electronic Science and Technology of China, Chengdu 611731, P.R. China.

[2] MOE Key Lab for Neuroinformation, High-Field Magnetic Resonance Brain Imaging Key Laboratory of Sichuan Province, University of Electronic Science and Technology of China, Chengdu 611731, P.R. China.

[3] Children's Hospital of Philadelphia, Department of Child and Adolescent Psychiatry and Behavioral Science, Philadelphia, PA USA.

[4] University of Pennsylvania, Department of Psychiatry, Philadelphia, PA USA.

[5] University of Cambridge, Department of Psychiatry, Cambridge CB2 0SZ, UK.

[6] Department of Medical Psychology, Chaohu Clinical Medical College, Anhui Medical University, Hefei 230032, P.R. China.

[7] School of Psychology, Southwest University, Chongqing 400715, P.R. China.

✉ Corresponding authors:

Huafu Chen (chenhf@uestc.edu.cn), and Wei Liao (weiliao.wl@gmail.com). The Clinical Hospital of Chengdu Brain Science Institute, MOE Key Laboratory for Neuroinformation, University of Electronic Science and Technology of China, Chengdu 611731, P.R. China. Fax: +86-28-61831273. Tel: +86-28-61831273.

CONTENTS

SUPPLEMENTAL RESULTS......3

1. QUALITY CONTROL 3
2. REPLICABLE MORPHOMETRIC SIMILARITY PATTERNS IN HC 5
3. THRESHOLD SELECTION IN CONSTRUCTION OF MSN OF HC 6
4. GLOBAL CASE-CONTROL MSN DIFFERENCES 8
5. REGIONAL MSN DIFFERENCES IN MDD RELATIVE TO HC...... 9
6. THRESHOLD SELECTION EFFECT ON CASE-CONTROL MSN DIFFERENCES 10
7. TIV EFFECT ON CASE-CONTROL DIFFERENCES 12
8. MEDICATION EFFECT ON CASE-CONTROL MSN DIFFERENCES......13
9. YEO FUNCTIONAL NETWORKS AND VON ECONOMO CLASSES 14
10. MSN VALUES-CLINICAL ASSOCIATIONS IN INDIVIDUALS WITH MDD......16
11. TRANSCRIPTOME ANALYSIS 18
    11.1 Gene expression of AHBA atlas *18*
    11.2 Estimation of regional gene expression *19*
    11.3 Enrichment pathways from significantly positively weighted genes *20*
    11.4 Multi-gene-list meta-analysis between MS differences-related genes and genes from GWAS *22*
12. VALIDATION ANALYSIS IN REPLICATION COHORT 24
    12.1 Reproducibility of MDD-related MS differences *24*
    12.2 Uncorrected overlapped ontology terms in reproducibility of MDD-related transcriptomic analysis *26*

SUPPLEMENTAL REFERENCES...... 27

Supplemental Results

1. Quality control

Participants were excluded for the following reasons from visual inspection of the images: failure of full cortical coverage during the DWI scan and/or having a poor-quality registration from DWI image to T1w image, and failing FreeSurfer surface segmentation. After quality control, 217 out of 242 MDD patients, and 208 out of 231 HC were included in the discovery cohort. In the replication cohort, 42 out of 51 MDD patients and 35 out of 43 HC remained. The detailed information was shown in Table S1. To check differences in motion and image quality between patients with MDD and HC, we calculated the Euler number for each T1w image. This approach was proposed by Rosen et al.[1] as a way to quantitatively assess image quality[2, 3]. Using a two-sided two-sample *t*-test, we found no significant differences in the Euler number between the two groups in discovery ($t_{(423)}$ = 0.70, p = 0.48) and replication ($t_{(75)}$ = 1.27, p = 0.21) cohorts (Figure S1).

Table S1. Clinical and Demographic Characteristics.

Variable	Discovery cohort MDD (n = 217)	Discovery cohort HC (n = 208)	*p*-values	Replication cohort MDD (n = 42)	Replication cohort HC (n = 35)	*p*-values
Demographic characteristic						
Age at scan, years	38.71 (11.95)	40.11 (15.52)	0.49[a]	39.02 (11.86)	34.03 (12.00)	0.07[a]
Female	143 (66%)	136 (65%)	0.91[b]	30 (71%)	26 (74%)	0.78[b]
Education, years	11.93 (3.52)	12.92 (3.80)	0.0009[a]	9.36 (4.59)	13.66 (3.94)	4.1E-05[c]
Clinical characteristic						
Duration, months	48.05 (65.86)	NA	NA	77.45 (94.42)	NA	NA
HAMD score	21.60 (5.05)	NA	NA	25.14 (5.23)	NA	NA
HAMA score	23.69 (8.32)	NA	NA	22.69 (7.05)	NA	NA
Medication, yes	(n = 209) 102 (47%)	NA	NA	39 (93%)	NA	NA

Note: Data are presented as either n (%) or means (standard deviations). The cohort size was obtained after the image data quality control.
Abbreviations: HC, healthy controls; HAMD, 17-item Hamilton Depression Scale; HAMA, Hamilton Anxiety Scale; NA, not available.
[a]Mann-Whitney *U*-test (two-sided). [b]Chi-square test. [c]Two-sample *t*-test (two-sided).

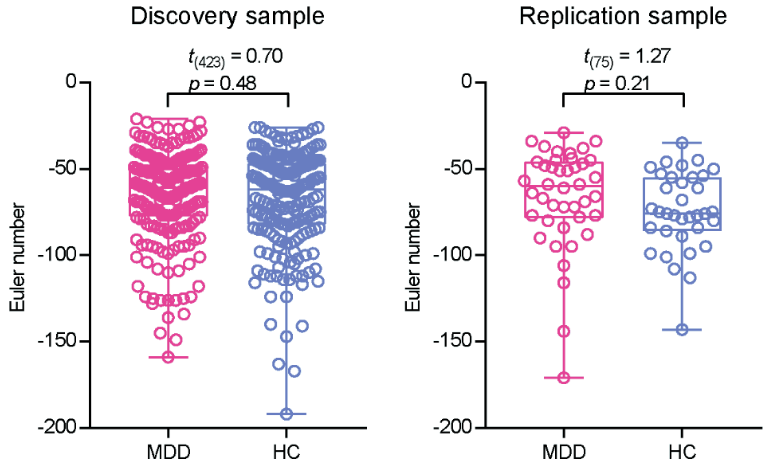

Figure S1. Euler number comparisons between MDD and HC, for both discovery and replication cohorts. Using a two-sample *t*-test, we found no significant differences in the Euler number between the two groups in discovery (MDD: n = 217; HC: n = 208; two-sample *t*-test: $t_{(423)} = 0.70$, $p = 0.48$) and replication (MDD: n = 42; HC: n = 35; two-sample *t*-test: $t_{(75)} = 1.27$, $p = 0.21$) cohorts. Boxplots show the lower quartile (25th percentile), median, and upper quartile (75th percentile), respectively. The upper and lower whiskers denote the minimum and maximum, respectively.

2. Replicable morphometric similarity patterns in HC

Figure S2 showed the relationship of MS pattern in HC between the current discovery cohort and Morgan *et al.* study (published online)[2]. These two MSN patterns were highly consistent (Pearson's $r_{(306)} = 0.91$, $p_{spin} < 0.0001$), with high positive morphometric similarity in frontal and temporal cortical areas and high negative morphometric similarity in occipital, somatosensory, and motor cortices, suggesting the robust and replicable regional MSN distribution.

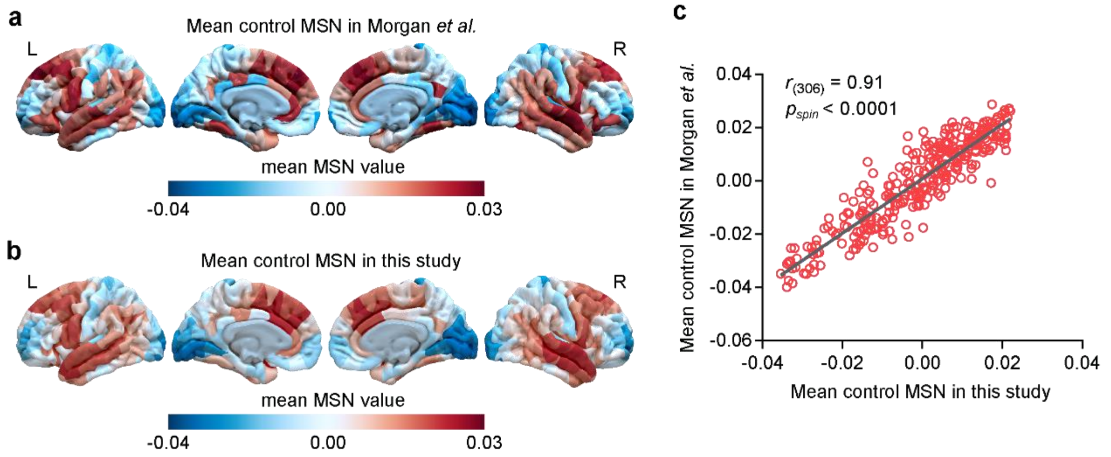

Figure S2. Replicable MS pattern in HC. a Regional MS averaged over HC in Morgan *et al.* study[3]. **b** Mean regional MS in HC in discovery cohorts of the present study. **c** Pearson's correlation analysis of regional MS between the two studies across regions (Pearson's $r_{(306)} = 0.91$, $p_{spin} = 0.00$). p value was determined based on a one-sided test.

3. Threshold selection in construction of MSN of HC

Threshold selection is a complex issue in network construction[3]. To validate the effects of thresholding on the MSNs, we calculated strength at a range of connection densities (10%–90% in 10% increments) generated by thresholding the MSNs. At all connection densities, the MSNs in HC demonstrated similar distributions of nodal strength with mean HC MSN based on non-thresholded networks; Figure 2a (Pearson's r values range from 0.406 to 0.998, all $p_{spin} < 0.0001$, FDR-corrected; Figure S3).

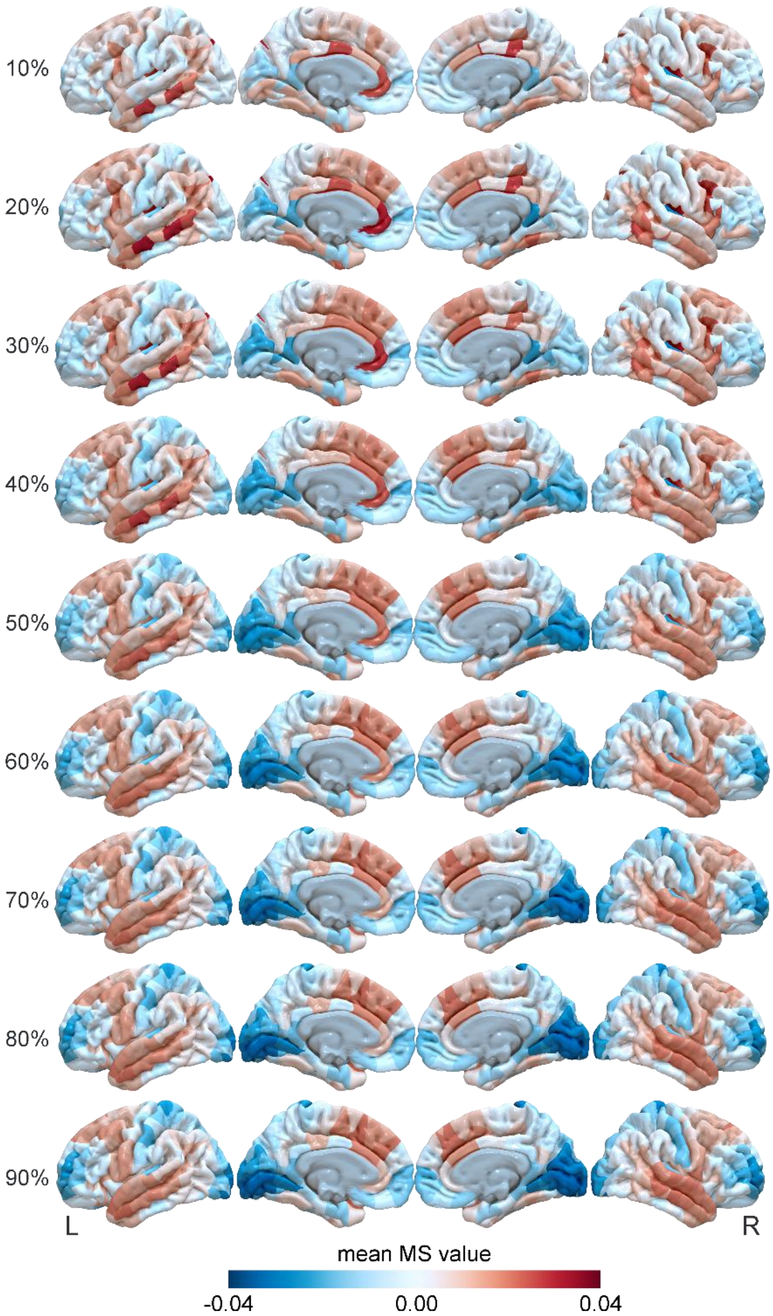

Thre	r	p_{spin}
10%	0.406	< 0.0001
20%	0.668	< 0.0001
30%	0.823	< 0.0001
40%	0.922	< 0.0001
50%	0.971	< 0.0001
60%	0.990	< 0.0001
70%	0.997	< 0.0001
80%	0.998	< 0.0001
90%	0.998	< 0.0001

Figure S3. Replicable MSNs across connection densities in HC. Left panel: Distributions of regional MS strength in HC at a range of connection densities (10%–90% in 10% increments). Right panel: Tabulated Pearson's correlation between mean MS values between non-thresholded MSNs and connection densities across parcels (10%: adjusted p_{spin} = 0.00; 20%: adjusted p_{spin} = 0.00; 30%: adjusted p_{spin} = 0.00; 40%: adjusted p_{spin} = 0.00; 50%: adjusted p_{spin} = 0.00; 60%: adjusted p_{spin} = 0.00; 70%: adjusted p_{spin} = 0.00; 80%: adjusted p_{spin} = 0.00; 90%: adjusted p_{spin} = 0.00). All p values adjusted by FDR, and were determined based on one-sided tests.

4. Global case-control MSN differences

Figure S4 plotted the global mean and distributions of regional MSN in discovery cohort.

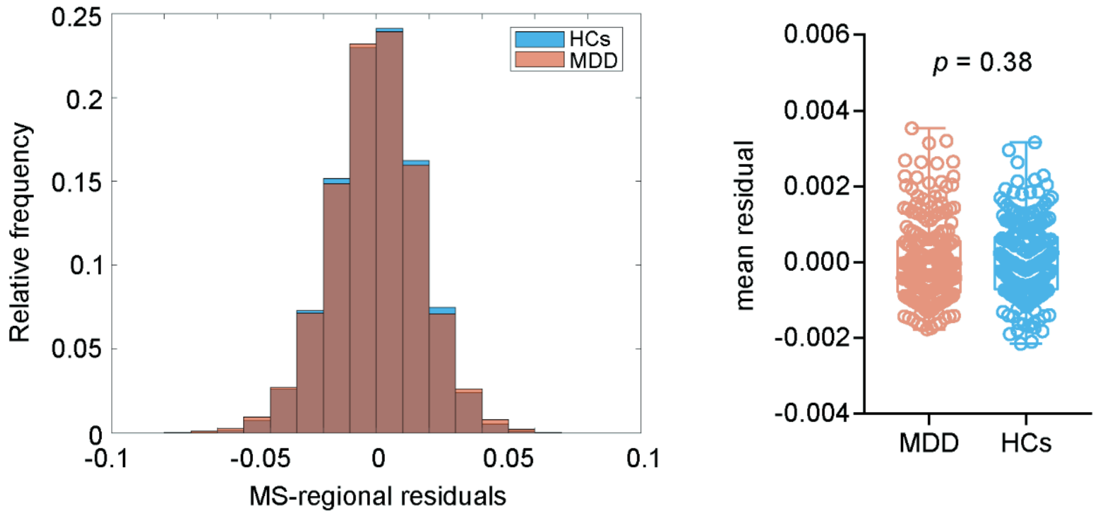

Figure S4. Global mean and distributions of regional MSN. Left panel: Distributions of regional MSN strength, i.e., the averaged MSN of each region with all other regions, after regressing age, sex, and education for individuals with MDD (red) and HC (blue). Right panel: Box plots for global mean MS (after regressing age, sex, and education) in patients with MDD (n = 217) and HC (n = 208). Using Mann-Whitney test, there are no difference between individuals with MDD and HC (U = 21467, p = 0.38, uncorrected, two-sided test). Boxplots show the lower quartile (25th percentile), median, and upper quartile (75th percentile), respectively. The upper and lower whiskers denote the minimum and maximum, respectively.

5. Regional MSN differences in MDD relative to HC

Seven brain regions showed statistically significant MS differences (all $p < 0.05$, FDR-corrected) in MDD relative to HC. The brain region' name, MNI coordinates, and statistic values of abnormal brain regions were listed in Table S2.

Table S2. Regions showing abnormal MSN in individuals with MDD

Region	MNI coordinates (x, y, z)			*t*-statistic	*p*-value
lh_isthmuscingulate_part2	-5.08	-42.79	28.46	3.36	0.0008
lh_lateraloccipital_part7	-28.70	-89.41	2.44	4.09	0.00005
lh_medialorbitofrontal_part2	-6.50	52.61	-11.10	3.33	0.0009
lh_superiorfrontal_part5	-10.35	54.69	27.23	-4.03	0.00006
rh_lateraloccipital_part1	18.65	-99.16	-7.39	3.29	0.001
rh_lateraloccipital_part6	32.22	-84.39	8.26	3.32	0.0009
rh_lateraloccipital_part8	29.43	-94.00	-5.36	4.10	0.00005

Note: Table giving details for the seven statistically regions anatomical labels, coordinates (in fsaverage, MNI305 space), *t*-statistic values (obtained by two-sample *t*-tests using linear regression model), and *p*-values. All *p*-values survived after FDR correction with $p < 0.05$, and were determined based on two-sided tests.

6. Threshold selection effect on case-control MSN differences

Although there was a minimal effect of threshold selection on MSN (validated in Supplemental Result 3), we also used additional connection densities (10%–90% in 10% increments) to investigate the stability of case-control MSN differences. At all connection densities, we found that the patterns of case-control MSN differences were similar to the equivalent non-thresholded MSNs; Figure 2b (Pearson's *r* values range from 0.270 to 0.999, all p_{spin} < 0.0001, FDR-corrected; Figure S5).

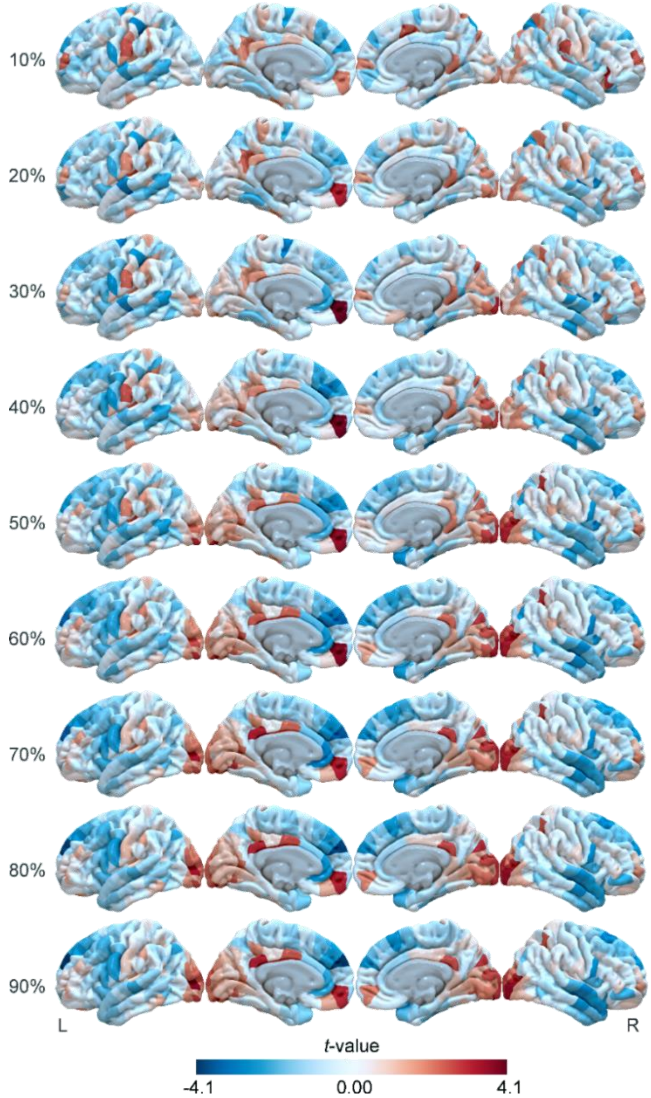

Thre	r	p_{spin}
10%	0.270	< 0.0001
20%	0.448	< 0.0001
30%	0.591	< 0.0001
40%	0.752	< 0.0001
50%	0.866	< 0.0001
60%	0.942	< 0.0001
70%	0.978	< 0.0001
80%	0.995	< 0.0001
90%	0.999	< 0.0001

Figure S5. Replicable case-control MSN differences across connection densities. Left panel: Distributions of MDD-related changes in MSN at a range of connection densities (10%–90% in 10% increments). Right panel: Tabulated Pearson's correlation coefficients between case-control t-maps with non-thresholded MSNs and connection densities (10%–90% in 10% increments) across parcels (10%: adjusted p_{spin} = 0.00; 20%:

adjusted p_{spin} = 0.00; 30%: adjusted p_{spin} = 0.00; 40%: adjusted p_{spin} = 0.00; 50%: adjusted p_{spin} = 0.00; 60%: adjusted p_{spin} = 0.00; 70%: adjusted p_{spin} = 0.00; 80%: adjusted p_{spin} = 0.00; 90%: adjusted p_{spin} = 0.00). All p values were adjusted by FDR, and were determined based on one-sided tests.

7. TIV effect on case-control differences

It is well-established that adjustment for total intracranial volume (TIV) can increase power in volumetric analyses[4]. Although there was no difference of TIV between MDD and HC (two-sided two-sample *t*-test, $t_{(423)} = 0.70$, $p = 0.49$) in the discovery cohort, we also validated the TIV effect on our case-control differences by including TIV as a covariate in the LRM. We found that the pattern of case-control differences was similar with our case-control differences without including TIV as a covariate (Pearson's $r_{(306)} = 0.997$, $p_{spin} < 0.0001$; Figure S6).

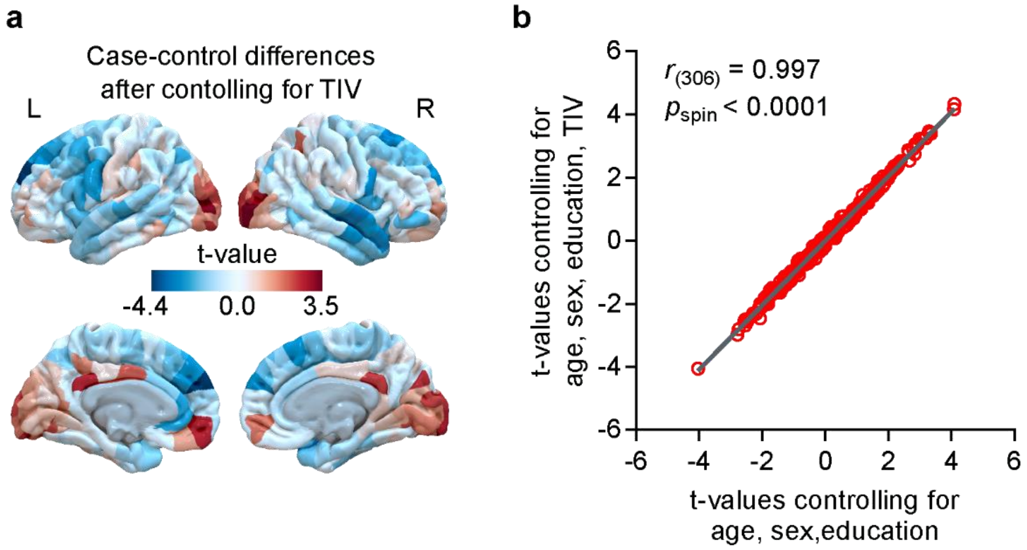

Figure S6. Validation of TIV effect on case-control MSN differences. a Regional case-control MSN differences after controlling for TIV in the discovery cohort. **b** Pearson's correlation for 308 t-statistic regional values for case-control differences with and without controlling for TIV (Pearson's $r_{(306)} = 0.997$, $p_{spin} = 0.00$). *p* value was determined based on a one-sided test.

8. Medication effect on case-control MSN differences

To investigate the effects of medication on case-control MSN differences, we divided the individuals with MDD into two subgroups: drug-experienced (n = 102) and drug-naïve (n = 107), where eight patients were without medication information and were thus excluded. For each group, we obtained two case-control MSN t-maps, drug-experienced vs. HC, and drug-naïve vs. HC, both of which showed similar case-control differences with the original combined group of individuals with MDD; Figure 2b (drug-experienced subgroup: Pearson's $r_{(306)}$ = 0.90, p_{spin} < 0.0001, FDR-corrected; drug-naïve subgroup: Pearson's $r_{(306)}$ = 0.89, p_{spin} < 0.0001, FDR-corrected; Figure S7).

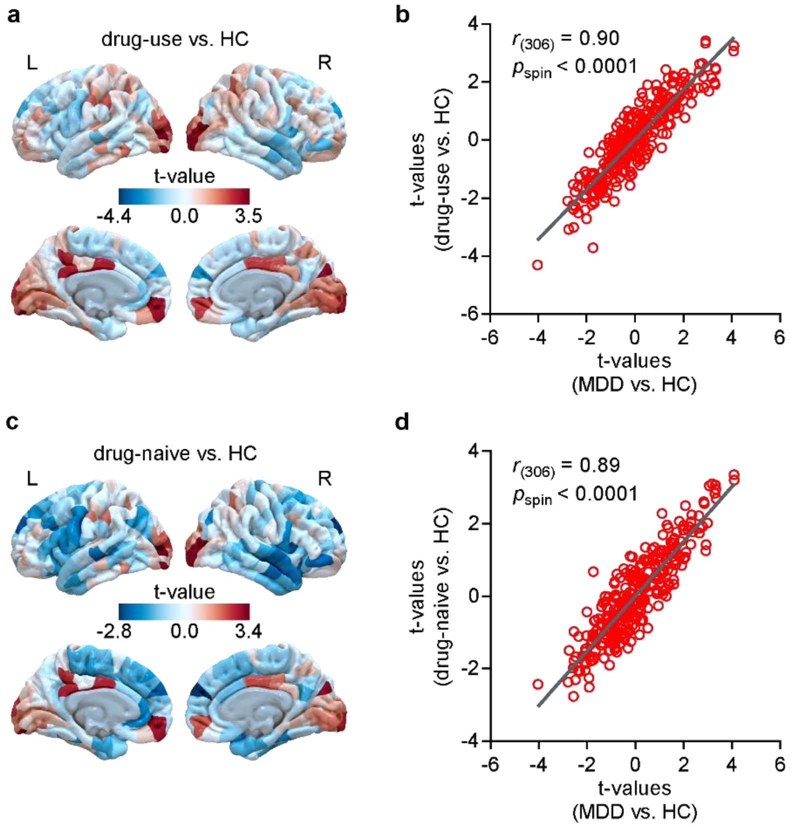

Figure S7. Medication effect on case-control MSN differences. a Regional *t*-map of drug-experienced group compared to HC in the discovery cohort. **b** Pearson's correlation for the 308 t-statistic values obtained from the drug-experienced vs. HC and MDD patients vs. HC. comparisons (Pearson's $r_{(306)}$ = 0.90, adjusted p_{spin} = 0.00, FDR-corrected). *p* value was determined based on a one-sided test. **c** Regional t-map of drug-naive compared to HC in the discovery cohort. **d** Pearson's correlation for the 308 t-statistic values obtained from drug-naive vs. HC groups and MDD patients vs. HC comparisons (Pearson's $r_{(306)}$ = 0.89, adjusted p_{spin} = 0.00, FDR-corrected). *p* value was determined based on a one-sided test.

9. Yeo functional networks and von Economo classes

To contextualize the regional MSN differences, we divided the all 308 cortical regions into two prior parcellation networks: Yeo 7 functional networks (Figure S8a) based on resting-state networks derived from functional MRI[5], and the von Economo classes of cortex (Figure S8b) classified by cytoarchitectonic criteria[6]. To perform this analysis, we calculated the *t*-statistics and corresponding *p* values for each network-related change in MSN within a particular Yeo 7 networks (Table S3) and von Economo class (Table S4). We found that individuals with MDD exhibited increased MSN in Yeo visual functional networks (p_{FDR} = 0.007) (Figure S8c). MSN was also increased in von Economo secondary sensory classes (p_{FDR} = 0.02) (Figure S8d).

Table S3. *t*-statistics and *p*-values for regional MSN differences across each Yeo network.

	Visual	Somatomotor	Dorsal attention	Ventral attention	Limbic	Frontoparietal	Default mode
t-statistic	3.290	-0.495	-0.575	-1.135	-0.711	-0.427	-2.008
p-value	**0.001**	0.621	0.565	0.257	0.478	0.670	0.048

Note: The abnormal networks were shown in bold font (Visual: p_{FDR} = 0.007) after FDR-corrected with $p < 0.05$. *t*-statistic values were obtained by two-sample *t*-tests. All *p* values were determined based on two-sided tests.

Table S4. *t*-statistics and *p*-values for regional MSN differences across each von Economo class.

	Primary motor	Association	Association	Secondary sensory	Primary sensory	Limbic	Insular
t-statistic	-1.815	-2.058	-1.231	3.408	1.186	1.431	0.139
p-value	0.070	0.044	0.219	**0.003**	0.236	0.153	0.889

Note: The abnormal classes were shown in bold font (Secondary sensory: p_{FDR} = 0.02) after FDR-corrected with $p < 0.05$. *t*-statistic values were obtained by two-sample *t*-tests. All *p* values were determined based on two-sided tests.

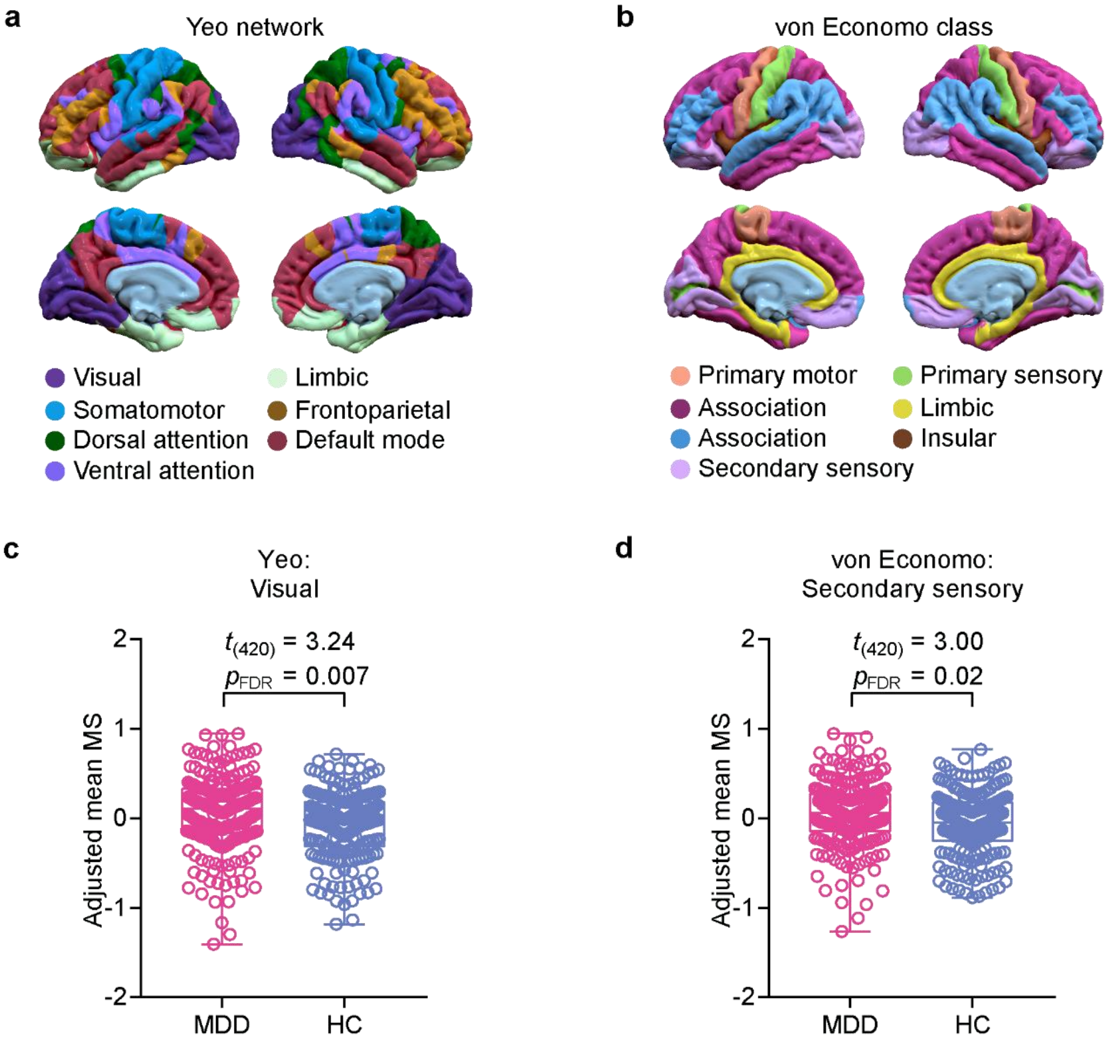

Figure S8. Yeo functional networks and von Economo classes of case-control differences in regional MSN. a The Yeo 7 functional networks. **b** The von Economo classes. **c** The distribution of case-control differences in Yeo-visual network (two-sample *t*-test: $t_{(420)}$ = 3.24, p = 0.001, two-sided test, FDR-corrected p_{FDR} = 0.007) between MDD (n = 217) and HC (n = 208) groups. **d** The distribution of case-control differences in von Economo classes-secondary sensory (two-sample *t*-test: $t_{(420)}$ = 3.24, p = 0.003, two-sided test, FDR-corrected p_{FDR} = 0.02) between MDD (n = 217) and HC (n = 208) groups. Boxplots show the lower quartile (25th percentile), median, and upper quartile (75th percentile), respectively. The upper and lower whiskers denote the minimum and maximum, respectively.

10. MSN values-clinical associations in individuals with MDD

We investigated the relationship between the regional MSN differences and clinical measures including 17-item Hamilton Depression Rating Scale (HAMD) and Hamilton Anxiety Rating Scale (HAMA). For each patient, we extracted the MS values in the 7 regions where MSN increased/decreased significantly in individuals with MDD compared to HC. The results of a Pearson's correlation between clinical measures and regional MSN values in the 7 regions are shown in Table S5. FDR correction was used to assess multiple comparisons.

We observed a positive correlation between MSN values of the right lateral occipital (part 8) and the HAMD scores (Pearson's $r_{(215)} = 0.135$, $p = 0.046$), which did not survive after FDR correction. This positive correlation meant that individuals with MDD with more severe HAMD scores tended to have higher degree of increased regional MSN differences compared to HC. The opposite direction was observed between MSN values of the left superior frontal (part5) and the HAMD scores (Pearson's $r_{(215)} = -0.117$, $p = 0.086$). There were no significant correlations between MSN values of significantly increased/decreased regions and the HAMA scores.

We also explored the relationship between MSN values and HAMD and HAMA scores at whole-brain level. As an exploratory analysis, we did not correct the *p*-values in correlation analysis. We found that the left DLPFC exhibited negative correlation with HAMD scores (Pearson's *r* values range from -0.14 to -0.22), whereas occipital cortices, middle/posterior cingulate cortex, and precentral cortex showed positive correlation with HAMD scores (Pearson's *r* values range from 0.14 to 0.20, Figure S9a). For HAMA scores, right DLPFC showed negative correlations (Pearson's *r* values range from -0.14 to -0.21), whereas left visual cortex and right temporal cortex exhibited positive correlations (*r* values range from 0.14 to 0.16, Figure S9b).

Table S5. The relationship between MSN values of abnormal regions and the HAMD and HAMA scores.

	Brain regions	HAMD		HAMA	
		r	*p*	*r*	*p*
Increased MSN	isthmuscingulate (part2, L)	0.129	0.059	-0.004	0.953
	lateraloccipital (part7, L)	0.087	0.204	-0.012	0.861
	medialorbitofrontal (part2, L)	0.017	0.802	0.085	0.215
	lateraloccipital (part1, R)	0.105	0.122	0.037	0.592
	lateraloccipital (part6, R)	0.121	0.074	0.050	0.459
	lateraloccipital (part8, R)	0.135	0.048	0.042	0.534
Decreased MSN	superiorfrontal (part5, L)	-0.117	0.086	-0.002	0.981

Note: All *r* values were obtained by Pearson's correlation analysis. All *p* values were not survived after FDR-corrected, and were determined based on two-sided tests.

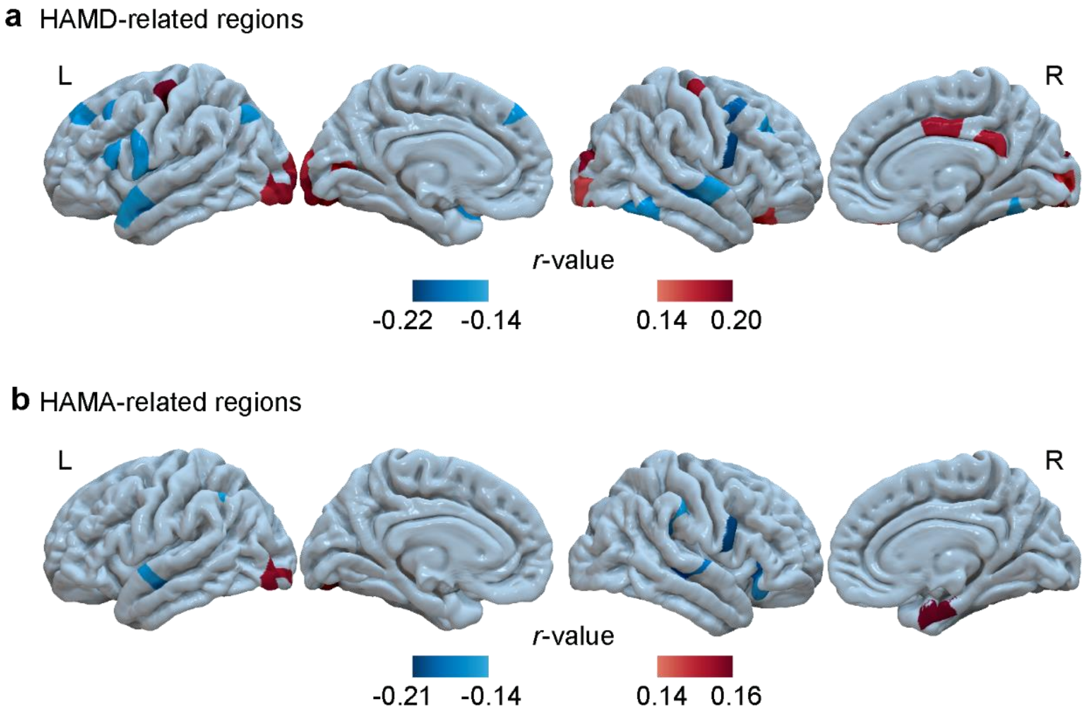

Figure S9. Correlation analysis between MSN values and clinical measures at whole-brain level. a MSN values related to HAMD scores. **b** MSN values related to HAMA scores.

11. Transcriptome analysis

11.1 Gene expression of AHBA atlas

We used the AHBA atlas (http://human.brain-map.org)—a whole-genome, whole-brain transcriptomic dataset to obtain brain gene expression. The AHBA dataset includes six neurotypical adult brains, three Caucasian, two African-American and on Hispanic. Their ages range from 24 to 57 years (age = 42.5 ± 13.38 years; male/female = 5/1). The detailed information was shown in Table S6.

Table S6. Demographics of six adult donors in AHBA dataset

Donor	Number of cohorts	Age	Sex	Ethnicity	Post-mortem interval [a]
H0351.2001[b]	946	24	Male	African American	23h
H0351.2002[b]	893	39	Male	African American	10h
H0351.1009	363	57	Male	Caucasian	25.5h
H0351.1012	529	31	Male	Caucasian	17.5h
H0351.1015	470	49	Female	Hispanic	30h
H0351.1016	501	55	male	Caucasian	18h

Note:

[a] Post-mortem interval is defined as the time period from the time of death to the time the tissue is frozen.

[b] These donors have tissue cohorts collected across the left and right hemispheres, while the other donors have tissue cohorts only in the left hemisphere.

11.2 Estimation of regional gene expression

We followed Arnatkevic *et al.*[7] to obtain brain gene expression: i) verifying probe-to-gene annotations. Probe-to-gene annotations were provided by Arnatkevic *et al.*[7] using Re-annotator toolkit[8]. After the reannotation, a final set of 45,812 probes were uniquely annotated to a gene and could be related to an entrez ID. Sequent analysis used the re-annotated set of 45,821 probes, corresponding to 20,232 unique genes; ii) filtering of probes that do not exceed background noise. To improve the validity of microarray expression measures, intensity-based filtering was used[9-11]. Here, probes that do not exceed the background in at least 50% of all cohorts across all subjects were excluded; iii) probe selection. Generally, multiple probes can be used to measure the expression level of a single gene at different exons, in these cases, probe with the highest correlation to RNA-seq data is selected[12]; iv) cohort assignment. To assign cohorts to brain regions more accurately, T1w of six donors' brains were firstly preprocessed using FreeSurfer. The D-K 308 atlas (152 regions in left hemisphere, and 156 regions in right hemisphere) employed in the neuroimaging dataset was reconstructed in each AHBA donor brain. Then a threshold was applied to avoid assigning cohorts beyond a certain distance[13], i.e., cohorts located less than 2 mm (Euclidean distance) to a region boundary were included; v) normalization of expression measures to account for inter-individual differences and outlying values. Gene expression data were normalized using the scaled robust sigmoid[14, 15] for each subject to eliminate the inter-individual differences in expression measures; vi) gene-set filtering. With the absence of a specific hypothesis, we selected genes based on differential stability to reduce donor-specific variance and focused on brain-relevant genes. Finally, the mean of all cohorts in a region was calculated to summarize the expression vector, resulting in a region × gene matrix of size 308 × 10,027. Because of AHBA dataset only includes two subjects with right hemisphere, we only considered gene expression in left hemisphere in our analysis, i.e., the region × gene matrix of size was 152 × 10,027.

11.3 Enrichment pathways from significantly positively weighted genes

As reported in the main test, there were 1,747 genes with $Z > 5$. The enrichment pathways of PLS+ genes were shown in Figure S10. The PLS1+ genes were enriched for GO biological processes, such as "signal release", and "synaptic vesicle priming", but no KEGG pathways. All these pathways were corrected by FDR, with *p* value shown in Table S7. The PLS1 gene names and *Z*-score weights were provided in Supplementary dataset 1.

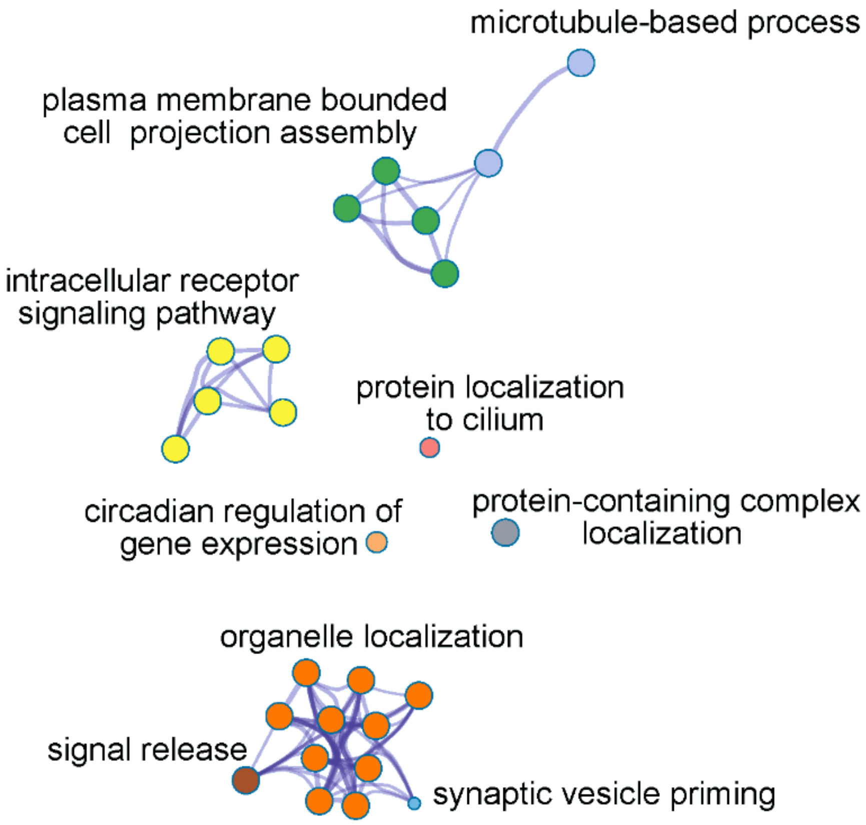

Figure S10. Enrichments pathways of positively weighted genes with $Z > 5$.

Table S7. GO Biological Process enrichments from the PLS+ gene set (*Z* > 5).

Category	*Z*-score	GeneInGOAndHitList	$-\log_{10}$(q-value)
GO: signal release	4.945	61	2.234
GO: organelle localization	5.12	88	2.617
GO: synaptic vesicle priming	5.675	8	1.743
GO: protein-containing complex localization	4.849	42	2.024
GO: plasma membrane bounded cell projection assembly	5.562	77	2.936
GO: microtubule-based process	4.287	87	1.752
GO: protein localization to cilium	5.075	14	1.752
GO: intracellular receptor signaling pathway	5.256	43	2.438
GO: circadian regulation of gene expression	4.957	15	1.706

11.4 Multi-gene-list meta-analysis between MS differences-related genes and genes from GWAS

We compiled data from two recent GWAS studies which provided genes that were significantly associated to the MDD phenotype[16, 17]. To specify the gene ranks obtained by the PLS method, we performed a multi-gene-list meta-analysis between PLS1- gene list and genes list from GWAS studies. Figure S11 showed the unique and overlapped enrichment pathways. The six overlapped same enrichment pathways between the two gene lists included “cognition”, “Ras protein signal transduction”, “regulation of ion transport”, “synaptic signaling”, “synapse organization”, and “cell-cell adhesion via plasma-membrane adhesion molecules”.

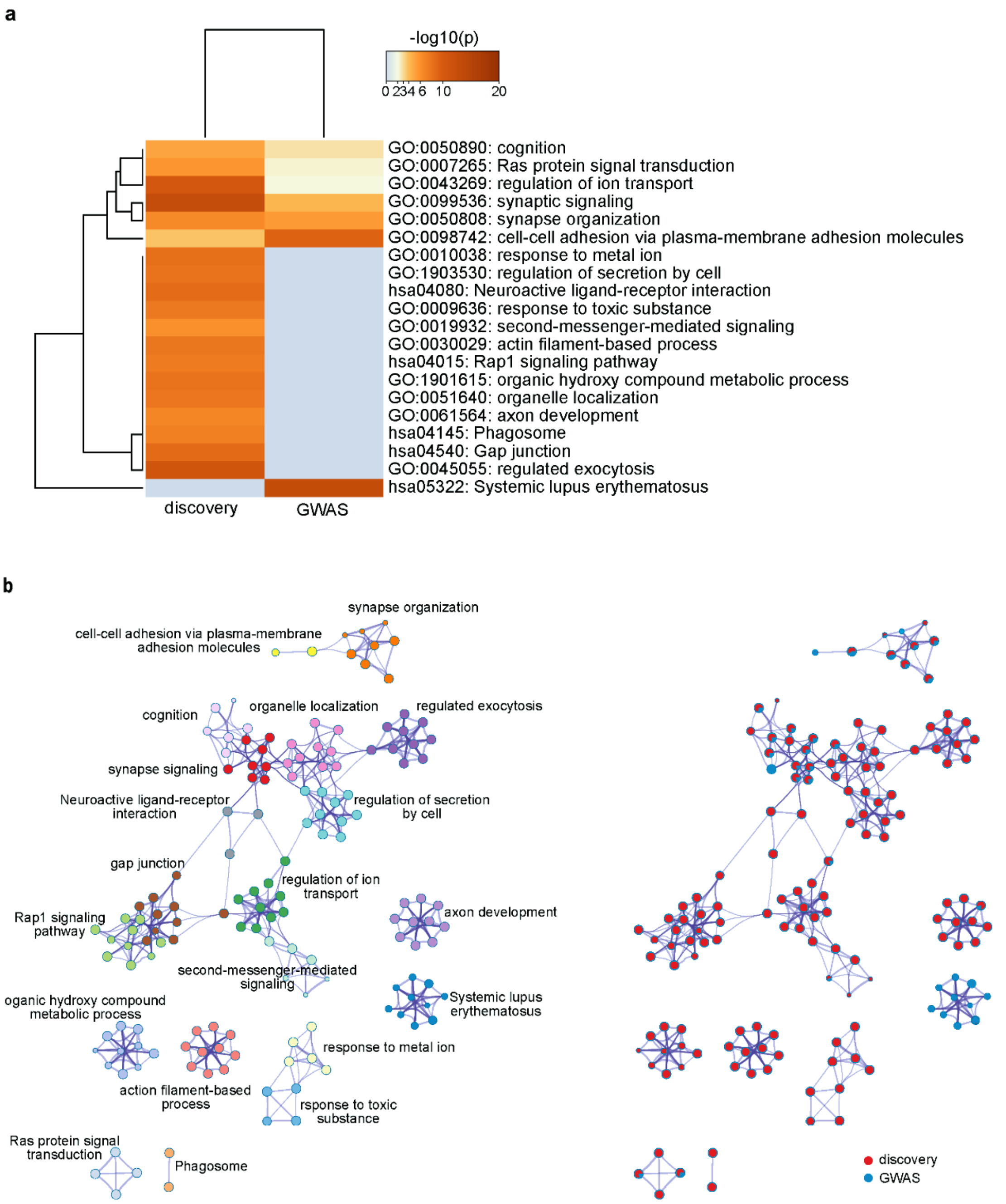

Figure S11. Overlapped ontology terms between discovery cohort and GWAS studies. (A) Enrichment heatmap visualization. The heatmap cells are colored by their p-values, white cells indicate the lack of enrichment for that term in the corresponding gene list. (B) Left panel was a subset of ontology terms. Right panel was the overlapped distribution. The same enrichment network has its nodes displayed as pies. Each pier sector is proportional to the number of hits originated from a gene list. Red is for discovery cohort, and blue is for GWAS studies.

12. Validation analysis in replication cohort

12.1 Reproducibility of MDD-related MS differences

Figure S12a showed the regional patient/control MSN *t*-statistics (after regressing out age, sex, and education levels) from replication cohort, with decreased frontal and temporal cortices and increased occipital cortex in MDD compared to HC. To observe the replicable MS differences, we performed the Pearson's correlation analysis for *t*-statistic values between discovery and replication cohorts (Figure S12b, Pearson's $r_{(306)}$ = 0.43, $p_{spin} < 0.0001$, this permutation testing based on spin of the spatially correlated MSN map) across regions. To validate not all *t* -maps of MSN differences might yield similar sized correlations, we performed an additional sensitivity analysis between case-control MSN *t*-map in discovery cohort and other conditions. Considering the common psychiatric and neurological disorders, we included a cross-sectional characterization of the MSN distribution of schizophrenia provided by a previous study (published online)[2], *t*-maps of epilepsy vs. control differences (our unpublished data), and male vs. females amongst HC participants in this study Together they represent, to our knowledge, the currently available MSN measurements. No significant correlations were found in these additional analyses (Figure S12c). In addition, the correlation of *t*-maps between discovery and replication cohorts was substantially higher than the correlation of *t*-maps between discovery cohort of MDD and schizophrenia vs. HC in Cobre center (Steiger's *z* value = 3.10, *p* = 0.002), epilepsy (Steiger's *z* value = 3.34, *p* = 0.0008) vs. HC and male vs. female (Steiger's *z* value = 3.94, *p* = 0.0001). These statistical comparisons of correlations were conducted in http://comparingcorrelations.org/[18].

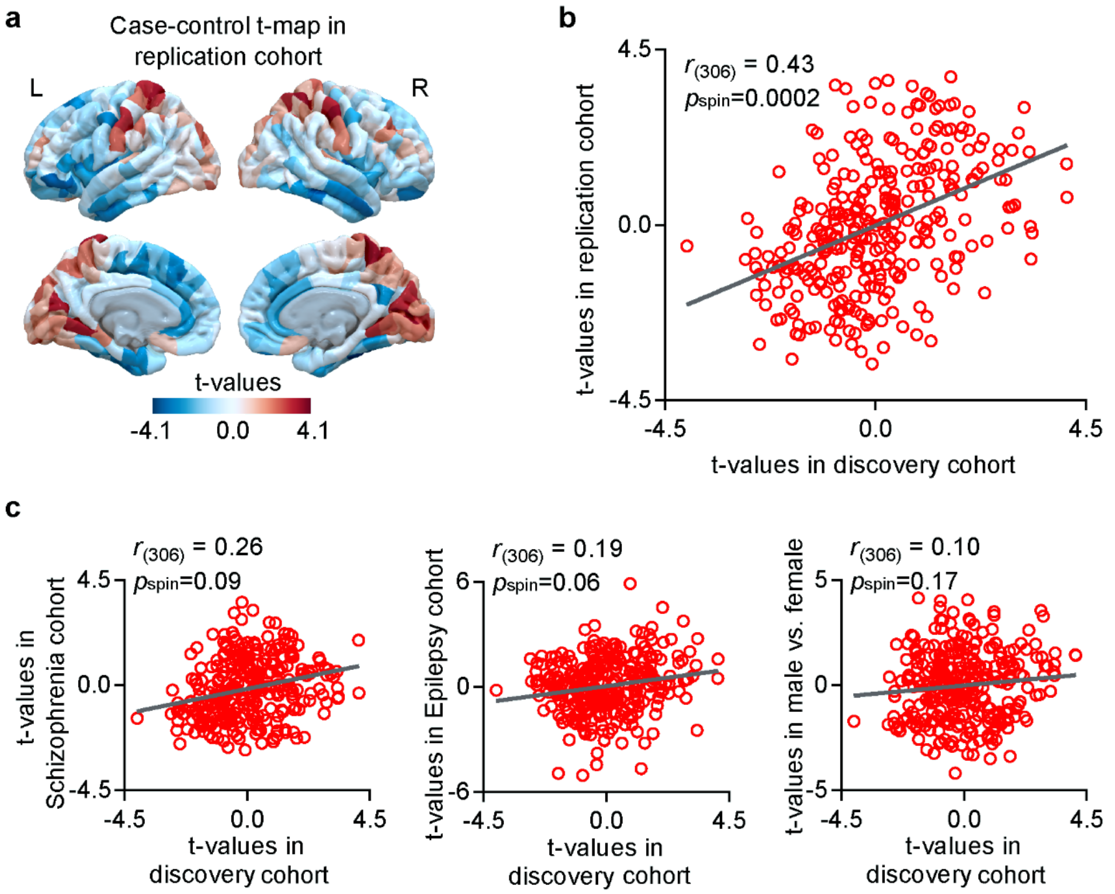

Figure S12. Replicable regional MSN differences of MDD. a Regional case-control MS differences in replication cohort. **b** Pearson's correlation analysis for 308 *t*-statistic values between discovery and replication cohorts (Pearson's $r_{(306)}$ = 0.43, p_{spin} = 0.0002). **c** Additional analyses for correlation of *t*-maps between discovery cohort and schizophrenia cohort (Pearson's $r_{(306)}$ = 0.26, p_{spin} = 0.09), epilepsy cohort (Pearson's $r_{(306)}$ = 0.19, p_{spin} = 0.06), and male vs. female (Pearson's $r_{(306)}$ = 0.10, p_{spin} = 0.17). All *p* values were not further corrected by multiple comparisons, and were determined based on one-sided tests.

12.2 Uncorrected overlapped ontology terms in reproducibility of MDD-related transcriptomic analysis

To our knowledge, this is the first study to explore the generalized ontology terms of regional MSN differences in individuals with MDD. Thus, Figure S13 showed the uncorrected overlapped ontology terms between discovery and replication cohorts for better visualization.

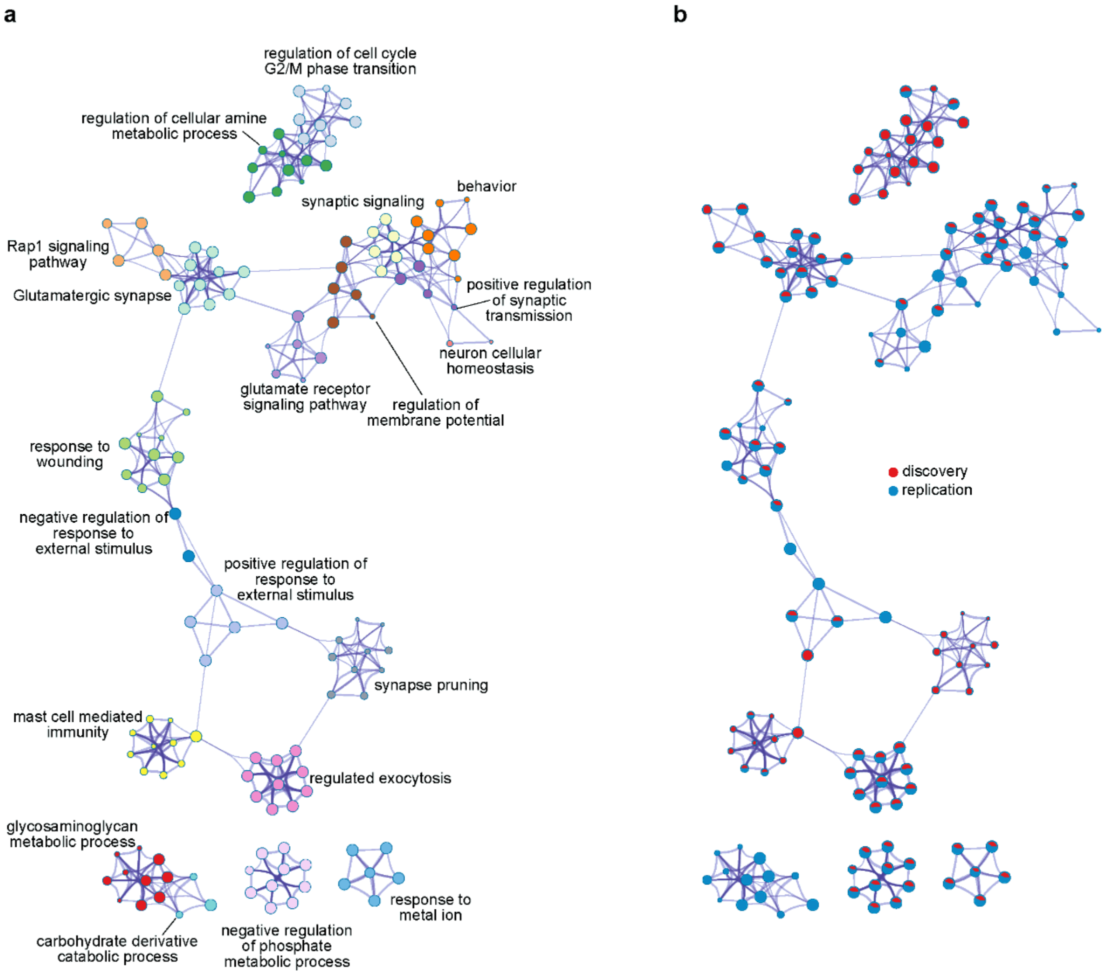

Figure S13. Uncorrected overlapped ontology terms between discovery and replication cohorts. a The name of overlapped ontology terms. **b** The same enrichment network has its nodes displayed as pies. Each pier sector is proportional to the number of hits originated from a gene list. Red is for discovery cohort, and blue is for replication cohort.

Supplemental References

1. Rosen, A. F. G. et al. Quantitative assessment of structural image quality. *Neuroimage* **169**, 407-418 (2018).
2. Morgan, S. E. et al. Cortical patterning of abnormal morphometric similarity in psychosis is associated with brain expression of schizophrenia-related genes. *Proc Natl Acad Sci U S A* **116**, 9604-9609 (2019).
3. Seidlitz, J. et al. Morphometric similarity networks detect microscale cortical organization and predict inter-individual cognitive variation. *Neuron* **97**, 231-247 e237 (2018).
4. Malone, I. B. et al. Accurate automatic estimation of total intracranial volume: a nuisance variable with less nuisance. *Neuroimage* **104**, 366-372 (2015).
5. Yeo, B. T. et al. The organization of the human cerebral cortex estimated by intrinsic functional connectivity. *J Neurophysiol* **106**, 1125-1165 (2011).
6. von Economo, C. & Koskinas, G. N. *Atlas of Cytoarchitectonics of the Adult Human Cerebral Cortex* (S Karger AG, 2008).
7. Arnatkevic Iute, A., Fulcher, B. D. & Fornito, A. A practical guide to linking brain-wide gene expression and neuroimaging data. *Neuroimage* **189**, 353-367 (2019).
8. Arloth, J. et al. Re-Annotator: Annotation Pipeline for Microarray Probe Sequences. *PLoS One* **10**, e0139516 (2015).
9. Hawrylycz, M. J. et al. An anatomically comprehensive atlas of the adult human brain transcriptome. *Nature* **489**, 391-399 (2012).
10. Burt, J. B. et al. Hierarchy of transcriptomic specialization across human cortex captured by structural neuroimaging topography. *Nat Neurosci* **21**, 1251-1259 (2018).
11. Richiardi, J. et al. BRAIN NETWORKS. Correlated gene expression supports synchronous activity in brain networks. *Science* **348**, 1241-1244 (2015).
12. Miller, J. A. et al. Transcriptional landscape of the prenatal human brain. *Nature* **508**, 199-206 (2014).
13. Romero-Garcia, R. et al. Structural covariance networks are coupled to expression of genes enriched in supragranular layers of the human cortex. *Neuroimage* **171**, 256-267 (2018).
14. Fulcher, B. D., Little, M. A. & Jones, N. S. Highly comparative time-series analysis: the empirical structure of time series and their methods. *J R Soc Interface* **10**, 20130048 (2013).
15. Fulcher, B. D. & Fornito, A. A transcriptional signature of hub connectivity in the mouse connectome. *Proc Natl Acad Sci U S A* **113**, 1435-1440 (2016).
16. Wray, N. R. et al. Genome-wide association analyses identify 44 risk variants and refine the genetic architecture of major depression. *Nat Genet* **50**, 668-681 (2018).
17. Howard, D. M. et al. Genome-wide meta-analysis of depression identifies 102 independent variants and highlights the importance of the prefrontal brain regions. *Nat Neurosci* **22**, 343-352 (2019).

18. Diedenhofen, B. & Musch, J. cocor: a comprehensive solution for the statistical comparison of correlations. *PLoS One* **10**, e0121945 (2015).
