# Supplementary material for: Cortical structural differences in major depressive disorder correlate with cell type-specific transcriptional signatures
Source: Nat Commun. 2021 Mar 12;12:1647. doi: 10.1038/s41467-021-21943-5 (PMC7955076; doi:10.1038/s41467-021-21943-5)
Supplement: Supplementary file 2 — Description of Additional Supplementary Files [file 41467_2021_21943_MOESM2_ESM.pdf]

Description of Additional Supplementary Files

Title: Supplementary Data 1

Description: The PLS1– and PLS1+ gene lists and Z-score weights from discovery cohort.
